# The synergistic interplay of artificial intelligence and digital twin in environmentally planning sustainable smart cities: A comprehensive systematic review

**DOI:** 10.1016/j.ese.2024.100433

**Published:** 2024-05-17

**Authors:** Simon Elias Bibri, Jeffrey Huang, Senthil Kumar Jagatheesaperumal, John Krogstie

**Affiliations:** a Swiss Federal Institute of Technology Lausanne (EPFL), Institute of Computer and Communication Sciences (IINFCOM), School of Architecture, Civil and Environmental Engineering (ENAC), Media and Design Laboratory (LDM), 1015, Lausanne, Switzerland; b Department of Electronics & Communication Engineering, Mepco Schlenk Engineering College, Sivakasi, 626005, Tamilnadu, India; c Department of Computer Science, Norwegian University of Science and Technology (NTNU), Trondheim, Norway

**Keywords:** Sustainable smart cities, Artificial intelligence, Artificial intelligence of things, Urban digital twin, Data-driven urban planning, Environmental planning, Environmental sustainability

## Abstract

The dynamic landscape of sustainable smart cities is witnessing a significant transformation due to the integration of emerging computational technologies and innovative models. These advancements are reshaping data-driven planning strategies, practices, and approaches, thereby facilitating the achievement of environmental sustainability goals. This transformative wave signals a fundamental shift — marked by the synergistic operation of artificial intelligence (AI), artificial intelligence of things (AIoT), and urban digital twin (UDT) technologies. While previous research has largely explored urban AI, urban AIoT, and UDT in isolation, a significant knowledge gap exists regarding their synergistic interplay, collaborative integration, and collective impact on data-driven environmental planning in the dynamic context of sustainable smart cities. To address this gap, this study conducts a comprehensive systematic review to uncover the intricate interactions among these interconnected technologies, models, and domains while elucidating the nuanced dynamics and untapped synergies in the complex ecosystem of sustainable smart cities. Central to this study are four guiding research questions: 1. What theoretical and practical foundations underpin the convergence of AI, AIoT, UDT, data-driven planning, and environmental sustainability in sustainable smart cities, and how can these components be synthesized into a novel comprehensive framework? 2. How does integrating AI and AIoT reshape the landscape of data-driven planning to improve the environmental performance of sustainable smart cities? 3. How can AI and AIoT augment the capabilities of UDT to enhance data-driven environmental planning processes in sustainable smart cities? 4. What challenges and barriers arise in integrating and implementing AI, AIoT, and UDT in data-driven environmental urban planning, and what strategies can be devised to surmount or mitigate them? Methodologically, this study involves a rigorous analysis and synthesis of studies published between January 2019 and December 2023, comprising an extensive body of literature totaling 185 studies. The findings of this study surpass mere interdisciplinary theoretical enrichment, offering valuable insights into the transformative potential of integrating AI, AIoT, and UDT technologies to advance sustainable urban development practices. By enhancing data-driven environmental planning processes, these integrated technologies and models offer innovative solutions to address complex environmental challenges. However, this endeavor is fraught with formidable challenges and complexities that require careful navigation and mitigation to achieve desired outcomes. This study serves as a comprehensive reference guide, spurring groundbreaking research endeavors, stimulating practical implementations, informing strategic initiatives, and shaping policy formulations in sustainable urban development. These insights have profound implications for researchers, practitioners, and policymakers, providing a roadmap for fostering resiliently designed, technologically advanced, and environmentally conscious urban environments.

## Highlights

• AI, AIoT, and UDT are reshaping the landscape of data-driven environmental urban planning.
• The synergistic interplay of AI, AIoT, and UDT is key to advancing sustainable smart cities.
• AI and AIoT augment UDT's capabilities to enhance data-driven environmental urban planning.
• Integrating AI, AIoT, and UDT in environmental planning presents formidable challenges.
• Both theoretical enrichment and valuable insights are offered to researchers and practitioners.

## Graphical abstract

**Figure:**
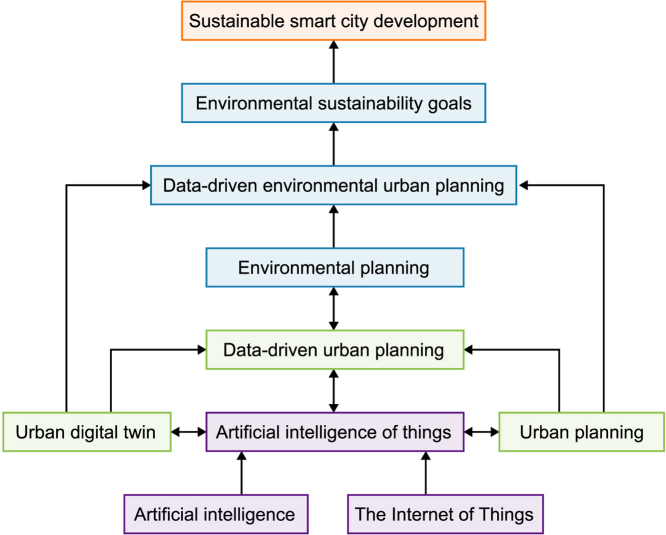

## Abbreviations

AAM: Advanced Air Mobility
AI: Artificial Intelligence
AIoDT: Artificial Intelligence of Digital Twin
AIoT: Artificial Intelligence of Things
CTIS: Cooperative Intelligent Transportation System
CNNs: Convolutional Neural Networks
CV: Computer Vision
DL: Deep Learning
DRL: Deep Reinforcement Learning
FL: Fuzzy Logic
IML: Interpretable Machine Learning
IoDT: Internet of Digital Twin
IoT: Internet of Things
LULC: Land Use and Land Cover
ML: Machine Learning
NLP: Natural Language Processing
PED: Positive Energy Districts
PRISMA: Preferred Reporting Items for Systematic Reviews and Meta-analyses
RL: Reinforcement Learning
SDGs: Sustainable Development Goals
SVR: Support Vector Regression
SVM: Support Vector Machine
UDT: Urban Digital Twin
XAI: Explainable Artificial Intelligence

## 1 Introduction

In recent years, the rapid pace of urbanization and technological advancement has accelerated the transformation of cities towards smarter and more sustainable pathways in response to the escalating complexity of ecological degradation and the pressing challenges of climate change. Central to this transformation are innovative technologies and models, notably artificial intelligence (AI), artificial intelligence of things (AIoT), and urban digital twin (UDT), which collectively offer unprecedented opportunities for advancing sustainable urban development and planning. The synergistic interplay of their applied data-driven solutions presents a powerful framework for advancing sustainable smart cities, facilitating informed decision-making, and fostering resilient urban ecosystems. However, to fully leverage their capabilities, a comprehensive understanding of their collaborative integration is imperative in the context of data-driven environmental planning in the urban landscape.

The convergence of AI, AIoT, UDT, data-driven urban planning, and environmental sustainability has emerged as a critical frontier in the development of sustainable smart cities. In recent years, AI and AIoT have significantly transformed data-driven urban planning practices, as evidenced by notable research contributions in the field [[1], [2], [3], [4], [5], [6]]. This integrated approach highlights the synergistic potential of AI and AIoT technologies in reshaping urban planning strategies to address complex environmental challenges. Indeed, these technologies have significantly impacted multiple urban domains by optimizing resource management, enhancing energy efficiency, maximizing the utilization of renewable energy sources, streamlining waste management processes, improving transportation systems, preserving biodiversity, reducing environmental footprints, and mitigating the potential risks of climate change [[7], [8], [9], [10], [11], [12], [13], [14], [15], [16]]. Through the provision of comprehensive environmental data, the facilitation of seamless integration of urban systems, and the coordination of various urban domains, AI and AIoT technologies closely align with the fundamental responsibilities of urban planners. The intelligence of AI lies in its capacity to learn from analysis results and outputs, a process that mirrors the fundamental logic of planning practice: gathering information, analyzing it, and producing plans or policies to address challenges and improve the quality of life [4]. In particular, AIoT is on the verge of significant transformations, poised to create more sustainable, efficient, resilient, and environmentally conscious urban environments.

Given the above, AI and AIoT have become foundational technologies in sustainable urban development. They have led to the emergence of the concepts of urban AI [17,18] and urban AIoT [8,19,20]. Urban AI integrates advanced AI techniques into urban systems, streamlining processes and augmenting decision-making capabilities. Conversely, urban AIoT synergizes AI capabilities with the Internet of Things (IoT) infrastructure, facilitating the creation of intelligent urban environments capable of real-time monitoring, analysis, and management. The amalgamation of urban AI and urban AIoT fuels innovation across various areas of urban planning and governance, providing effective solutions to multifaceted urban challenges [8,19]. By leveraging data-driven insights, predictive modeling, optimization techniques, community engagement, and adaptive strategies, urban planners can harness AI and AIoT to create more sustainable, resilient, and inclusive cities that meet the evolving needs of citizens and stakeholders.

Sustainable smart cities are increasingly embracing and leveraging AI and AIoT technologies [7,19,[21], [22], [23], [24]], represent dynamic ecosystems that demands a fundamental reevaluation of their current planning approaches to tackle complex environmental challenges. The ongoing transformation of sustainable smart cities highlights the imperative for a profound shift in understanding and planning urban environments. At the heart of this transformation lies the concept of UDT — a dynamic virtual replica of a real-world city's physical, spatial, and functional aspects, mirroring its structures, systems, and dynamics in real-time. UDT, a tool for simulating urban environments and developing scenarios for planning and policy problems [25,26], enables innovative approaches and reinforces strategies for tackling the environmental challenges faced by sustainable smart cities [19,27,28]. It is a powerful tool for local governments, allowing them to simulate “what-if” scenarios and potential solutions to address diverse urban conditions. UDT facilitates identifying and implementing targeted intervention measures to advance environmental sustainability goals by enabling analysis, visualization, and response to these conditions. Consequently, its adoption is rising globally, with examples found in various regions and cities, especially in emerging sustainable smart cities.

By leveraging smart IoT sensors, advanced data analytics, powerful AI algorithms, and innovative visualization methods, UDT integrates vast and diverse data from multiple sources to facilitate real-time monitoring and improve predictions and decision-making in urban planning. Indeed, AI and AIoT technologies have recently found their way into the computational functionalities of UDT [29,30], enriching data-driven environmental planning initiatives [[31], [32], [33], [34], [35], [36]] in sustainable smart cities. By integrating AI models, such as machine learning (ML), deep learning (DL), computer vision (CV), and natural language processing (NLP), planners can effectively manage vast datasets, identify patterns, and discern trends via UDT systems, thereby facilitating more informed decision-making across various domains through automation, optimization, and prediction.

To understand the significance of AI and AIoT in data-driven environmental planning in the context of sustainable smart cities, it is important to trace the trajectory of urbanization and the evolutionary path of planning models. Environmental planning involves assessing, managing, and optimizing the use of natural resources and the environment to foster sustainable development, minimize environmental impact, and enhance the well-being of communities. Smart cities, as hubs for technological advancements, have experienced remarkable developments and concurrent challenges. In this dynamic landscape, the emergence and adoption of data-driven technologies, spurred by the imperative for sustainable development, have given rise to the concept of sustainable smart cities [21,[37], [38], [39], [40]]. This emerging urbanism paradigm emphasizes environmental sustainability across various domains (see Ref. [7] for a bibliometric analysis and detailed review). At the nexus of these transformative trends, integrating AI and AIoT with UDT in sustainable smart cities is rapidly evolving, merging their physical, spatial, functional, digital, and computational realms to elevate their environmental performance.

The focal point of this study revolves around the multifaceted contributions of UDT, facilitated by the capabilities of AI and AIoT, to data-driven environmental planning in sustainable smart cities. The synergistic interplay of AI, AIoT, and UDT fosters a dynamic relationship where each component influences and complements the other. With its advanced algorithms and data processing capabilities, AI harnesses insights from vast UDT data generated via IoT devices to optimize decision-making processes. AIoT enhances the capabilities of IoT devices through AI algorithms, enabling them to adapt, learn, and optimize their performance autonomously. Conversely, UDT provides AI with rich, real-world context and spatial data, enhancing the accuracy and relevance of AI-driven analyses and predictions. UDT integrates real-time data from IoT devices with AI-driven simulations to predict and evaluate various scenarios for data-driven environmental planning. Planners can also explore ways to optimize the collection and configuration of data generated through their planning efforts, leveraging AI analyses to facilitate more effective data-driven and evidence-based decision-making processes to achieve better sustainability outcomes [4,5]. The synergistic interplay enables more precise environmental assessments, scenario simulations, and proactive sustainable urban development strategies, empowering stakeholders to make informed decisions for creating resilient and environmentally conscious cities. By integrating AI and AIoT capabilities with UDT frameworks, urban planners can harness the power of advanced data analytics, predictive modeling, and real-time monitoring to optimize environmental strategies.

While the scholarly landscape has primarily explored urban AI, urban AIoT, and UDT in isolation, a significant knowledge gap exists regarding their synergistic interplay, collaborative integration, and collective impact on data-driven environmental planning in the dynamic context of sustainable smart cities. To address this gap, this study conducts a comprehensive systematic review to uncover the intricate interactions among these interconnected technologies, models, and domains while elucidating the nuanced dynamics and untapped synergies in the complex ecosystem of sustainable smart cities. The study formulates the following four research questions (RQs) to guide the comprehensive systematic review.

RQ1: What are the theoretical and practical foundations underpinning the convergence of AI, AIoT, UDT, data-driven planning, and environmental sustainability in sustainable smart cities, and how can these components be synthesized into a novel comprehensive framework?
RQ2: How does integrating AI and AIoT reshape the landscape of data-driven planning to improve the environmental performance of sustainable smart cities?
RQ3: How can AI and AIoT augment UDT's capabilities to enhance data-driven environmental planning processes in sustainable smart cities?
RQ4: What challenges and barriers arise in integrating and implementing AI, AIoT, and UDT in data-driven environmental urban planning, and what strategies can be devised to surmount or mitigate them?

Guided by these research questions, the study aims to comprehensively understand the current research landscape and its evolving dynamics. It offers invaluable insights into the transformative potential of integrating AI, AIoT, and UDT to advance data-driven environmental planning in sustainable smart cities. These insights, combined with foundational knowledge, can serve as a roadmap to guide future investigations, strategic initiatives, practical implementations, policy formulations, and innovative approaches to sustainable urban development.

The remaining sections of this study are structured as follows: Section 2 outlines and justifies the methodology employed in the study. Section 3 presents the results, addressing the four research questions posed earlier. Section 4 provides a detailed discussion encompassing an interpretation of results, a comparative analysis, the implications, the limitations, and suggestions for future research. Finally, Section 5 summarizes the study's key findings and contributions.

## 2 Materials and methods

This study presents a comprehensive systematic review, examining the intricate interplay of AI, AIoT, UDT, data-driven urban planning, and environmental sustainability, as well as the nuanced dynamics and untapped synergies in the dynamic landscape of sustainable smart cities. The current scholarly landscape is inherently fragmented, primarily because the convergence of these technologies, models, and domains is relatively new — yet rapidly evolving. The topics are emerging and coming together, and as research progresses and technologies advance over time, themes will become evident. A thorough systematic review of a novel landscape of research and practice featuring emerging topics and themes serves several important purposes. One of these is to ascertain the current state-of-the-art knowledge in this rapidly evolving interdisciplinary field. This enables researchers to acquire a more profound understanding of emerging discoveries, transformations, innovations, and findings, laying the groundwork for more extensive explorations and empirical investigations. Another purpose is establishing the context and relevance of emerging topics and themes. Researchers can better grasp their significance in the field by contextualizing the new interconnected technologies, models, and domains in the broader research domain.

In this systematic review, the preferred reporting items for systematic reviews and meta-analyses (PRISMA) approach was employed for literature search and selection [41]. This approach is a widely accepted framework designed to enhance systematic reviews' transparency, consistency, rigor, and comprehensiveness. It encompasses several stages, including defining eligibility criteria for study selection, developing a thorough literature search strategy, selecting relevant studies, extracting data, appraising the quality of studies, and analyzing and synthesizing the extracted data. Adhering to the PRISMA approach ensured a high level of methodological rigor in the comprehensive systematic review, enhancing the findings' reliability and validity. Furthermore, this approach facilitated the identification of emerging patterns, trends, and gaps in the existing body of literature, ultimately contributing to a more comprehensive understanding of the multifaceted research topic at hand.

**Fig. 1:**
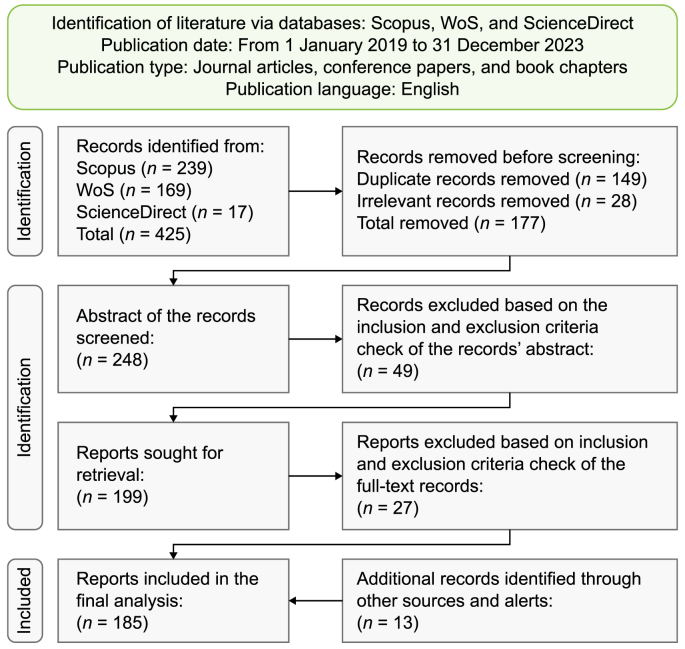
The PRISMA flowchart for literature search and selection. Adapted from Page et al. [41].

Fig. 1 illustrates the three-phase flowchart associated with the PRISMA approach. From the array of bibliographic databases accessible, Scopus, Web of Science (WoS), and ScienceDirect were chosen due to their extensive coverage of the high-quality peer-reviewed studies relevant to the multifaceted topic at hand. The number of records identified was found to overlap across these databases. Employing a thorough search strategy to retrieve the relevant scholarly literature, a set of pertinent keywords was carefully selected based on the combination of sustainable smart cities, UDT, AI, AIoT, urban planning, and environmental sustainability. The search string encompassed vital combinations such as “sustainable smart cities AND artificial intelligence OR artificial intelligence of things,” “sustainable smart cities AND urban digital twin,” “sustainable smart cities AND urban planning,” “sustainable smart cities AND environmental sustainability AND artificial intelligence,” “urban digital twin AND artificial intelligence OR artificial intelligence of things,” “urban digital twin AND urban planning,” “urban digital twin AND environmental sustainability,” and “urban digital twin AND urban planning AND environmental sustainability.” These were used to search against the title, abstract, and keywords of documents to produce initial insights. These combinations ensured specificity, diversity, and relevance in searching and retrieving the sought data.

The inclusion criteria filtered studies based on pertinence, reliability, language, publication date, and publication type (article, conference paper, or book chapter), providing definitive primary information. Exclusion criteria were applied to remove studies unrelated to the focal topics and their interlinkages and irrelevant to the research aims and questions. Based on these predefined inclusion/exclusion criteria, the selection process involved initial screening based on titles and abstracts, followed by a detailed full-text review for eligibility. The search query retrieved 425 records from three databases (Scopus = 239, WoS = 169, and ScienceDirect = 17). After removing duplicates, 149 records were eliminated. Subsequently, titles and keywords were scrutinized, leading to the exclusion of 28 more records. The remaining 248 records underwent abstract screening against the inclusion and exclusion criteria, removing 49. The full-text screening of the remaining 199 records led to excluding an additional 27 records. Ultimately, this process yielded a final selection of 172 publications. Additionally, 13 extra records were included through other sources and alerts, bringing the total number of records included in the final analysis to 185. Throughout the process, a critical appraisal was conducted to assess the quality of the selected studies. The search process encompassed various peer-reviewed articles, conference proceedings, and book chapters. The objective was to establish a nuanced understanding of the multifaceted research topic, gain deeper insights into theoretical frameworks and contextual landscapes, identify potential opportunities and synergies, and anticipate challenges and barriers along with related mitigation strategies.

The literature search for this comprehensive systematic review was conducted in early September 2023, aiming to capture the latest developments in the dynamic field of sustainable smart cities. The chosen time frame spans from January 2019 to December 2023 to ensure a comprehensive review of recent literature, aligning with the rapid advancements and emerging trends in the specified technological areas of AI, AIoT, and UDT in relation to data-driven urban planning and environmental sustainability domains. This period allowed us to encapsulate the latest five years of scholarly contributions, providing up-to-date analysis and synthesis of knowledge and exploring the most contemporary perspectives on the multifaceted interplay of these technologies, models, and domains. However, it was observed that fewer studies were published in 2019 compared to subsequent years. This lower number of studies in 2019 could be attributed to several factors related to the topic of the study and evolving research trends. One possible explanation is that the topic experienced increased research interest in the years following 2019, leading to more studies being conducted and published. Additionally, changes in research priorities and technological advancements may have influenced the publication landscape, with researchers shifting focus to explore emerging areas of interest.

Guided by an inductive approach, a content analysis was performed on the included studies to gather the data needed to analyze and synthesize the existing literature. The obtained insights enabled the identification of key themes, patterns, and variations, ensuring a comprehensive analysis and synthesis. A quality assessment was performed to ensure the credibility and reliability of the selected studies, contributing to drawing meaningful conclusions related to the technological convergence and synergistic integration in question. Particularly, peer-reviewed studies from reputable sources were given precedence, and an evaluation was applied to ascertain the validity and robustness of the methodologies employed in each study by assessing the strengths and weaknesses of the research design. Overall, the guided extraction of insights enabled the identification of key themes, patterns, variations, and insightful nuances, ensuring a comprehensive data analysis. This process included breaking down the data into distinct parts, assigning appropriate labels, identifying connections and relationships between these labels, and developing central themes derived from the identified labels. Throughout this phase, a continuous comparison of new data with existing labels and categories was conducted to ensure a thorough exploration. Subsequently, the data synthesis phase encompassed integrating, interpreting, and developing overarching themes from the analyzed data. This entailed categorizing information based on commonalities and differences, formulating conceptual and descriptive categories that encapsulated the essence of the data, and constructing a narrative that interwove key findings and insights.

Specifically, the comprehensive systematic review combined configurative, aggregative, and narrative synthesis techniques. Configurative synthesis was utilized to explore the foundational theories and practical applications that underpin the convergence of AI, AIoT, UDT, data-driven planning, and environmental sustainability in the context of sustainable smart cities. Aggregative synthesis techniques were employed to analyze how the integration of AI and AIoT influences data-driven planning, reshaping urban landscapes to enhance the environmental performance of sustainable smart cities. They were also used to identify how AI and AIoT augment the capabilities of UDT to advance data-driven environmental planning processes in sustainable smart cities. A narrative synthesis was adopted to analyze and interpret the findings gathered from the comprehensive systematic review process. This synthesis technique involved organizing and summarizing the key themes, concepts, and insights extracted from the literature on AI, AIoT, UDT, data-driven planning, and environmental sustainability in the context of sustainable smart cities. By synthesizing these findings narratively, the study provided a coherent and nuanced understanding of the synergistic effects of these technologies and models on data-driven environmental urban planning. Narrative synthesis was instrumental in weaving together the findings from the configurative and aggregative synthesis stages, allowing for the development of a balanced narrative that synthesized the diverse perspectives and insights uncovered throughout the comprehensive systematic review process. It facilitated the elucidation of the intricate interplay of AI, AIoT, UDT, data-driven planning, and environmental sustainability, providing a holistic understanding of their integrated implications for sustainable urban development.

## 3 Results: analysis and synthesis

This section presents the results of the comprehensive systematic review, providing insights into the synthesized evidence across 185 studies. It focuses on the theoretical and practical foundations underlying this convergence, the transformative impact of AI and AIoT integration on the landscape of data-driven urban planning to enhance environmental sustainability outcomes, and how the AI- and AIoT-augmented UDT advance data-driven environmental urban planning processes. Lastly, attention is given to the challenges and barriers inherent in the integration and implementation of AI, AIoT, and UDT in data-driven environmental urban planning, along with strategies to surmount or mitigate them.

### 3.1 Theoretical and practical foundations

This section analyzes and synthesizes the existing literature on the theoretical and practical foundations underlying the convergence of UDT, AI, AIoT, data-driven urban planning, and environmental sustainability in the dynamic context of sustainable smart cities. It examines the concept of sustainable smart cities, shedding light on the evolving landscapes influenced by urban AI, urban AIoT, and UDT and their effects on the changing dynamics of urban planning in terms of its data-driven and environmental dimensions. This sets the stage for a comprehensive exploration of the contributions made by emerging urban computing, modeling, and planning paradigms to advancing environmental goals in sustainable smart cities.

**Fig. 2:**
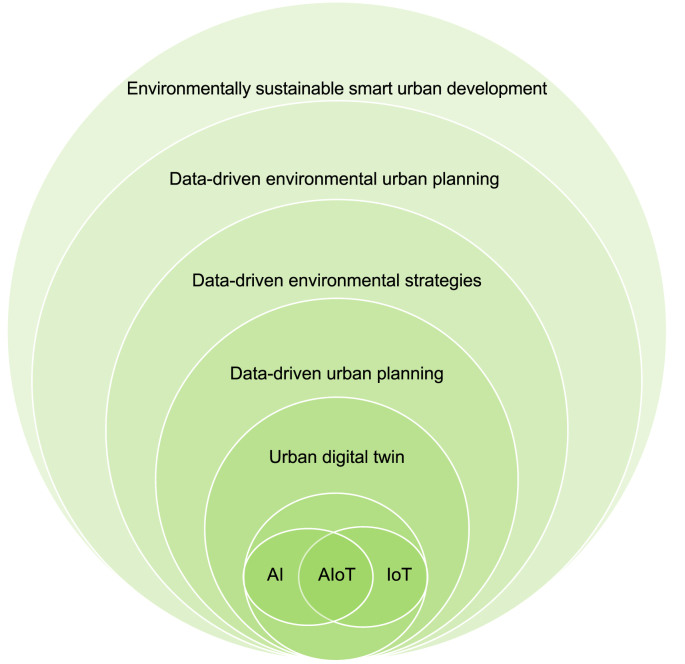
An overview of the primary conceptual and practical foundations identified along with their interconnections.

Fig. 2 illustrates key technologies, models, and domains and their interconnections in a circular form from a hierarchical perspective. The relationship among AI, AIoT, UDT, data-driven urban planning, environmental strategies, data-driven environmental planning, and environmentally sustainable urban development goals is symbiotic, dynamic, and multifaceted. AI and AIoT technologies are key enablers in the data-driven urban planning process, providing advanced analytics and insights from vast amounts of data generated by IoT devices embedded in urban environments. UDT systems further enhance this process by creating detailed virtual replicas of sustainable smart cities, enabling real-time monitoring, simulation, and optimization of various systems in these cities. These technologies and models, when integrated, facilitate the development and implementation of data-driven environmental planning strategies aimed at addressing challenges such as energy usage, waste generation, pollution, resource depletion, and climate change. By leveraging AI and AIoT capabilities within UDT frameworks, urban planners and policymakers can make informed decisions, optimize resource allocation, and design interventions to achieve environmentally sustainable urban development goals, ultimately leading to more efficient, resilient, and livable cities.

#### 3.1.1 Sustainable smart cities

Sustainable smart cities refer to urban environments that leverage advanced technologies and data-driven solutions to enhance efficiency, improve the quality of life, and address environmental challenges while ensuring long-term viability. Emerging in response to the criticism directed at traditional smart city initiatives [[42], [43], [44], [45], [46]], sustainable smart cities are increasingly integrating data-driven technologies with a focus on achieving the sustainable development goals (SDGs) [[47], [48], [49]]. Ahad et al. [50] comprehensively reviewed the enabling technologies for smart cities, identified key challenges encompassing technical, socioeconomic, and environmental dimensions, and proposed best practices for realizing sustainable smart cities. The study conducted by del Mar Martínez-Bravo and Labella-Fernández [51] aims to elucidate the concept of “sustainable smart cities” and propose a broader understanding. It advocates for sustainability-coherence, limited growth, heightened awareness, and combining bottom-up and top-down initiatives as essential characteristics of sustainable smart cities. Evaluating their strengths and weaknesses becomes paramount as sustainable smart city projects proliferate globally. Hence, there is a need to develop assessment tools to provide essential performance indicators for multiple stakeholders engaged in urban development.

Transitioning from smart to sustainable smart cities necessitates a holistic approach to sustainability, with a heightened emphasis on environmental and technological factors. The existing body of literature provides valuable insights into pathways for fostering more intelligent and environmentally conscious urban futures. Sustainable smart cities exemplify the integration of technological advancements and environmental strategies, leveraging AI and AIoT solutions for renewable energy, energy conservation, water resource management, waste management, sustainable transportation, pollution control, climate change mitigation, and intelligent infrastructure [7,8]. Numerous studies have explored the roles of not only AI and AIoT but also UDT in advancing environmental sustainability through innovative approaches to planning and management in sustainable smart cities [[7], [8], [22], [23], [24], [27], [28], [52]].

By assimilating the latest advancements and cutting-edge technologies, sustainable smart cities can evolve into dynamic change agents capable of responding to the growing needs of their citizens, the dynamics of their environment, and the mounting pressures of urbanization. The defined principles and practices of sustainable smart cities provide a foundational blueprint for navigating the complex landscape of environmental sustainability. They exemplify the collaborative integration of UDT with AI and AIoT to shape urban environments that are technologically advanced and environmentally resilient, empowered by sophisticated data-driven planning systems.

#### 3.1.2 Urban digital twin

UDT represents a sophisticated technological concept reshaping sustainable urban development's landscape. It represents a detailed and dynamic virtual replica that mirrors the city's physical, functional, spatial, and behavioral aspects in real-time or near-real-time, providing a dynamic platform for analysis, planning, and decision-making. The primary goal of UDT is to serve as a powerful tool for enhancing urban planning, management, and governance processes and practices to advance progress toward SDGs [[53], [54], [55], [56]], with a particular emphasis on attaining the environmental objectives of sustainable smart cities [27,28]. UDT, continuously updated with real-time data from IoT sensors and devices and analyzed using AI models and techniques [29,30,57], enables city planners and stakeholders to monitor, model, simulate, and visualize complex urban systems, understand behavioral patterns, test scenarios, predict environmental changes, optimize urban infrastructure design, and enhance services.

The multifaceted and crucial role of AIoT in UDT lies in integrating AI computational and analytical functionalities into IoT devices across urban infrastructures. This enables real-time data acquisition, analysis, and prediction and empowers IoT systems and applications with intelligence and decision-making capabilities. This integration plays a pivotal role in establishing a dynamic and responsive framework for smarter, more resilient, and sustainable urban ecosystems that improve the quality of life for citizens while minimizing environmental impact.

Drawing insights from real-world implementation in various sustainable smart cities worldwide, UDT has demonstrated its capacity to provide a detailed and interactive platform tailored for diverse data-driven planning and design functions [[57], [58], [59], [60], [61], [62], [63]], including.

• Simulation and modeling: UDT enables accurate and dynamic three-dimensional (3D) city simulations based on spatial, environmental, socio-economic, historical, and real-time data. This allows planners to model and test scenarios and potential solutions to assess their impact and effectiveness.
• Data-informed decision-making: It supports data-driven decision-making by extracting meaningful insights based on integrated data. Planners can analyze trends and patterns to make more informed choices in sustainable urban development.
• Collaborative planning: It fosters collaboration among stakeholders, including city planners, architects, engineers, community members, and citizens, in a virtual environment, promoting inclusive and participatory planning.
• Resource allocation optimization: It provides insights into the efficiency of urban infrastructure, energy and material usage, and transportation systems, optimizing resource allocation for overall sustainability.
• Resilience planning enhances urban resilience by allowing planners to simulate and plan for natural disasters, climate change, and emergency response strategies.
• Monitoring and management: Real-time UDT monitoring enables proactive urban service management. Planners can promptly identify and respond to issues, ensuring efficient city infrastructure operation.

UDT is a virtual testing ground for urban planning strategies and decisions. It leverages advanced technologies to create a comprehensive and dynamic representation of a city, enabling more effective, adaptive, data-driven, and collaborative approaches to sustainable urban development.

#### 3.1.3 Urban artificial intelligence

AI is the simulation of human intelligence processes by computer systems or machines. These processes include perception, learning, reasoning, problem-solving, decision-making, and language understanding. AI systems are designed to perform complex tasks that typically necessitate human-like intelligence, including visual perception, object detection, speech recognition, data pattern recognition, emotion detection, discernment of communication behavior, and making predictions and decisions based on complex datasets. AI can be characterized as the ability to achieve objectives in diverse and uncertain environments using highly adaptive, general-purpose systems through self-directed learning [64]. While humans may automate tasks manually, AI can reliably and efficiently execute high-volume tasks autonomously [65].

AI encompasses a wide variety of models and technologies, including ML, DL, CV, NLP, Evolutionary Computing (EC), and robotics. These models have been applied to various urban planning and design functions [[1], [2], [3], [4], [5], [6]] in the context of environmental sustainability in sustainable smart cities [7,8,21,22,[24], [27], [66], [67]]. The integration of AI technologies into urban planning and design processes underscores the pivotal role of AI in advancing the development of sustainable smart cities. At the heart of this integration lies the overarching goal of AI: to develop systems capable of autonomous task performance and adaptive learning. This collective human-like intelligence function is central to the application of AI in sustainable smart cities, where AI systems are harnessed to optimize urban planning and design processes to address complex challenges.

In light of the above, urban AI, as an interdisciplinary field, explores how AI technologies can be applied to urban contexts. It aims to enhance urban planning, design, management, governance, and decision-making processes by providing insights, predictions, and recommendations to improve cities' efficiency, resilience, and livability [8,19]. Cugurullo et al. [68] explored AI's multifaceted impact on urban environments, highlighting the co-constitutive relationship between AI and modern cities. Cugurullo et al. [17] critically assessed the transformation of urbanism due to AI, highlighting its distinct theoretical and practical implications for urban development. The authors argued that AI urbanism, influenced by AI, presents a new paradigm potentially leading to post-smart cities. They compared smart urbanism with AI-driven urbanism, discussing limitations and potentials while providing conceptual tools for understanding its impact. Palmini and Cugurullo [18] focused on the intersection of AI urbanism and sustainability, critiquing the notion of sustainable AI urbanism and proposing a redefined framework. The authors advocated for a shift towards sustainability in AI urbanism by reexamining fundamental ideas and design cultures. Palmini and Cugurullo [69] charted the landscape of AI urbanism by examining its conceptual sources and spatial implications. The authors provided a framework for understanding the AI-urbanism relationship, emphasizing the need for both qualitative and quantitative approaches and considering the material impact of AI on cities for fostering sustainable urban innovation. Overall, urban AI intersects with data-driven environmental planning by leveraging advanced computational techniques and large datasets to analyze urban environments and their ecological impact. Urban AI can process vast amounts of data from various sources to model and predict environmental trends. By integrating AI-driven insights into environmental planning processes, decision-makers can make more informed decisions to promote sustainability, resilience, and human well-being in urban areas.

#### 3.1.4 Urban artificial intelligence of things

In the rapidly changing landscape of technological innovation, the theoretical foundation and symbiotic interaction of AI and IoT encapsulated in the concept of AIoT stand as core pillars shaping the trajectory of sustainable smart cities. AIoT, as a technological framework, harnesses the complementary strengths and capabilities of AI and IoT to reshape urban landscapes [8,[13], [15],^19^,^70^]. It entails incorporating AI techniques and algorithms, especially ML and DL, into IoT devices to enhance functionality and augment intelligence [13,[15], [71]]. AIoT aims to make IoT devices collect and transmit data and analyze and interpret these data using AI tools, thereby making IoT systems more adaptive, efficient, and capable of autonomous decision-making. This goal is achieved through digital instrumentation, digital hyper-connectivity, datafication, algorithmization, and platformization [72]. Parihar et al. [73] explore IoT growth, AI integration benefits, AIoT architecture, applications, and implementation challenges.

Urban AIoT is about integrating AI and IoT technologies in urban environments to analyze, understand, and improve urban systems and processes and coordinate urban domains and networks. It involves deploying AI algorithms and techniques to analyze vast data collected from urban infrastructures and systems and diverse IoT devices embedded throughout the city's environment. This encompasses data from transportation systems, energy grids, water and waste management systems, smart buildings, public safety sensors, environmental monitoring devices, and other urban IoT deployments [8,10,[13], [14], [15], [16], [74]]. Urban AIoT aims to enhance cities' efficiency, sustainability, and livability by enabling intelligent decision-making, predictive analytics, and process automation across diverse domains. This integration allows cities to leverage real-time data insights to optimize resource allocation, improve service delivery, and address urban challenges effectively.

It is crucial to highlight the growing body of research exploring the transformative potential of AI and AIoT in promoting environmental sustainability and addressing climate change risks in the context of sustainable smart cities. While previous studies have examined aspects of AI and AIoT in these individual domains [9,12,75], the comprehensive systematic review by Bibri et al. [8] stands out for its extensive analysis of applied AI and AIoT solutions for environmental sustainability and climate change mitigation and adaptation in emerging smarter eco-cities. This review analyzes and synthesizes a diverse and large body of research, covering the multifaceted dimensions of the integration of eco-urbanism, smart urbanism, and AI or AI-driven urbanism, thereby providing a holistic understanding of environmentally sustainable smart urban development.

Intelligent technology, propelled by the synergies of AI and AIoT principles, is poised to revolutionize the framework of sustainable smart cities. Recent advancements and cutting-edge resources underscore the collaborative impact of AI and AIoT in optimizing urban operational functioning and planning. Integrating AI and IoT technologies lays the groundwork for developing more sustainable cities and heralding a new era of urban intelligence. In this context, AIoT catalyzes cities to leverage networked devices and smart algorithms for informed and data-driven decision-making. The synergistic interplay and distinctive character of AI and AIoT emerge as crucial elements shaping the broader vision of intelligent and resilient urban areas, charting the crossroads of technology and sustainability.

#### 3.1.5 Urban planning

Urban planning is a multidisciplinary and complex process encompassing designing, regulating, and managing land use and resources in urban areas and their connecting infrastructure. Its overarching goal is to enhance the overall quality of life, effectively address the challenges of urbanization and ecological degradation, and contribute to achieving SDGs. It encompasses various conceptual domains, commonly classified into different types, including strategic planning, sustainable planning, land use planning, transportation planning, local and regional planning, infrastructure planning, and environmental planning [76]. These conceptual areas address diverse aspects of sustainable urban development. However, urban planning grapples with the intricate challenges arising from the dynamic nature of urban environments. Characterized by the complexity of multidimensional and systemic factors, urban planning confronts what is frequently referred to in policy analysis as “wicked problems” [77]. These typically involve multiple stakeholders, tangled interdependencies, and unpredictable outcomes. In essence, wicked problems demand holistic, adaptive, and collaborative strategies to traverse their complex nature and find sustainable solutions.

Batty and Marshall [78], Marshall [79], and Portugali [80] explored the complexity of urban systems and their implications for urban planning. They highlighted the shift from traditional top-down planning approaches to a bottom-up perspective, emphasizing cities' dynamic and continually evolving nature. Additionally, they discussed the challenges posed by complexity, including the unknowability of urban systems and the difficulty of predicting their future states. These studies advocate for a more nuanced understanding of urban complexity and adopting planning strategies that embrace and harness complexity to generate functional urban complexity. Bibri [76] built on these insights by examining the role of data-driven technologies in planning sustainable smart cities. The author argued that sustainable cities are inherently complex systems and proposed a framework for data-driven sustainable smart cities. This framework leverages urban computing and intelligence to analyze and optimize urban systems to improve sustainability, efficiency, resilience, equity, and quality of life. By integrating complexity, sustainability, and data-intensive sciences, the study contributed to advancing the planning and design of sustainable cities by harnessing the power of big data and emerging technologies.

Bettencourt [81] discussed the utility of big data in urban planning by framing the planning process as a broad computational problem. The author illustrated that by aligning new data sources with urban policies under general conditions, one can apply fundamental engineering and applied science principles to devise novel, more efficient solutions for urban problems. The study argued that big data is pivotal in facilitating information flows and channels, coordinating mechanisms, fostering cooperative communication, and supporting learning and sharing processes among diverse constituents and heterogeneous collective and individual actors acting as data agents. However, it is also highlighted that irrespective of the abundance of available data, achieving a comprehensive form of urban planning remains computationally challenging, especially in large cities. Nevertheless, as suggested by Sanchez [4], a practical approach to envisioning the integration of AI into sustainable planning practice involves identifying strategic points of intervention that align with typical planning tasks: (a) community visioning, (b) plan-making, (c) standards, policies, and incentives, (d) development work, and (e) public investments. Planners can analyze the tasks associated with each point and consider how AI could automate or enhance these processes.

In the contemporary urban development landscape, the imperative for a data-driven approach to planning arises from the need to address complex and dynamic challenges facing cities. Conventional urban planning often has inefficiencies, uncertainties, and a lack of real-time insights. Integrating advanced technologies and digital models offers a transformative solution by infusing data-driven methodologies into planning.

#### 3.1.6 Data-driven urban planning

Urban planning has undergone a transformative shift, adopting a dynamic, data-driven approach that extends beyond traditional methods, propelled by cutting-edge approaches and innovative strategies. Data-driven urban planning heavily relies on analyzing, interpreting, and utilizing various forms of data to inform decision-making processes. It focuses on understanding urban dynamics, identifying patterns, and predicting future trends, a practice associated with UDT as a data-driven urban planning system [19]. This approach leverages data-driven technologies to aid urban planners in developing more informed and effective strategies for improving urban infrastructure, enhancing public services, and addressing sustainability and resilience challenges [[81], [82], [83]]. Komninos et al. [84] extended the prevailing theory of urban development to smart city planning, exemplifying a strategy emphasizing economic, environmental, and social sustainability. This approach represents a departure from traditional planning paradigms, emphasizing the integration of technologies, active user engagement, and the exploration of emerging opportunities, reflecting the evolving nature of cities. The endeavor to construct cities characterized by resilience, adaptability, functionality, and livability requires a strategic orchestration of land use, infrastructure, services, and more, with advanced technologies playing a key role.

In the last decade, the landscape of data-driven planning in smart cities has witnessed the exploration of diverse advanced methods, with a predominant reliance on big data analytics and IoT [[85], [86], [87], [88], [89], [90], [91]]. Recent research particularly underscores the pivotal role of AI in urban planning, with real-time data, advanced data analytics models, AI algorithms, and spatial analysis emerging as integral tools [2,[6], [92]].

In the realm of sustainable smart cities, data-driven urban planning has evolved into a dynamic process that harnesses the latest AI and AIoT models and techniques to envision cities attuned to the needs of citizens and responsive to environmental challenges. This process increasingly intertwines with AI, IoT, AIoT, and UDT. AI-driven decision support systems enhance the efficiency and effectiveness of urban planning strategies. IoT contributes to data-driven urban planning by connecting physical objects and infrastructure to the internet, allowing real-time data collection and monitoring [85,89,90]. IoT sensors placed throughout the city can capture information on various environmental parameters. These real-time data feed into decision-making processes enabled by AI for optimizing urban systems and enhancing the immediacy of information available to city planners [4]. AIoT further enriches data-driven urban planning, enabling real-time monitoring, efficient resource utilization, advanced analytics, predictive modeling, adaptive approaches, and more intelligent decision-making and automation. As regards UDT, which incorporates AI, IoT, and AIoT into its functionalities [19,29,30], it contributes to data-driven planning by facilitating simulations, scenario forecasting, and visualization of urban systems and dynamics for decision-making purposes. Integrating AI, IoT, AIoT, and UDT transforms traditional urban planning into a dynamic, data-driven, comprehensive approach. This empowers city planners with actionable insights for informed decision-making to build smarter and more sustainable cities.

#### 3.1.7 Environmental planning

Environmental planning plays a crucial role in ensuring the sustainable development of urban areas, addressing key challenges related to resource management, pollution control, and ecosystem preservation. Ndubisi [93] defined it as “the process of understanding, evaluating, and providing options for the use of landscape to ensure a better fit with human habitation.” Environmental planning is a means to mediate the dialogue between human activities and natural processes, drawing upon an understanding of the reciprocal interaction between people and the environment [94]. It entails a comprehensive framework of methodologies, plans, and strategies that revolve around evaluating, managing, and optimizing natural resources and ecosystems. This multifaceted approach is designed to foster sustainable development, mitigate environmental footprints, and improve the overall well-being of communities [95]. Mersal [96] provided an overview of the importance of environmental/ecological planning in sustainable urban development and proposes a conceptual framework for developing sustainable urban ecosystems. Ndubisi [93] examined different historical and comparative approaches to this planning process, highlighting their distinct perspectives on the interaction between human activities and the environment. In relevance to this study, environmental planning aims to create environmentally responsible, resource-efficient, and resilient cities to climate change. This aligns with smarter eco-cities, which advocate for promoting efficient public transportation, energy optimization, waste reduction, water conservation, biodiversity protection, pollution control, and climate change mitigation [8].

In recent years, the adoption of advanced technologies has emerged as a transformative force in environmental planning, offering innovative tools and methods to tackle complex challenges [[97], [98], [99]] and forge a path towards sustainable smart cities. Data-driven technologies, especially AI and AIoT, play a key role in improving the efficiency and effectiveness of environmental planning processes and practices. This entails harnessing state-of-the-art tools, such as ML/DL models, expert systems (ES), decision support systems (DSS), geographic information systems (GIS), remote sensing, big data analytics, and planning support systems (PSS) [75,92,100]. For example, studies on biodiversity and ecosystem services have utilized ML techniques for modeling competition and population dynamics and especially modeling ecosystem services, while species conservation efforts have benefited from ML and DSS [[101], [102], [103]]. These technological advancements enable planners to gather, analyze, and interpret vast amounts of environmental data with heightened levels of performance and speed. Essentially, converging environmental planning and advanced technologies represent a powerful synergy. By harnessing the capabilities of cutting-edge tools, planners can unravel the complexities of sustainable development more effectively, ensuring that environmental considerations are at the forefront of decision-making processes.

#### 3.1.8 Data-driven environmental sustainability strategies

In sustainable smart cities, environmental sustainability strategies entail plans or actions designed to ensure that urban activities are carried out in a manner that preserves and protects the natural environment in urban areas. Examples of these strategies include renewable energy adoption, energy efficiency improvements (e.g., buildings, transportation, and infrastructure), waste reduction and recycling programs, green space preservation and expansion, water conservation measures, transportation policies, green building standards, and climate mitigation and resilience planning. Environmental sustainability strategies in smart sustainable cities are increasingly shaped by data-driven approaches and AI/AIoT solutions to tackle complex environmental challenges [8,22,23,27,104]. These data-driven strategies leverage various data sources, including sensor data, satellite imagery, historical records, and more, to analyze environmental impact, track progress, and optimize resource management. Data-driven approaches, along with AI/AIoT, play a crucial role in enhancing the environmental performance of smart sustainable cities. They include smart energy management, smart grids, traffic management, waste management, water management, air quality monitoring, green space management, pollution control, carbon footprint tracking, disaster management, circular economy initiatives, and urban planning (see Refs. [7,8] for detailed reviews). They rely heavily on data, AI algorithms, and AIoT tools to understand current environmental conditions, predict future trends, and make informed decisions to minimize environmental impact while promoting sustainability in smart cities and beyond [[9], [10], [11], [12], [13], [14],[15], [16], [75]]. In the context of sustainable smart cities, AIoT can enhance environmental sustainability strategies by enabling real-time monitoring, analysis, and optimization of resource use.

Based on data-driven strategies, planners can gain deeper insights into and address environmental challenges, pinpoint areas for improvement, and devise targeted interventions by harnessing data from diverse sources. For example, data on air quality, water usage, waste generation, and transportation can inform decisions on infrastructure development and resource management to advance environmental sustainability goals. Moreover, data-driven environmental sustainability strategies are embedded in UDT platforms to monitor and assess the environmental performance of sustainable smart cities [19,28]. In this context, urban planners can test strategies for reducing carbon emissions, improving energy efficiency, and enhancing ecosystem resilience before implementation by simulating the impact of different environmental policies and interventions. Overall, environmental sustainability strategies are closely linked with data-driven urban planning, UDT, and AIoT in sustainable smart cities, which can integrate these approaches to develop more effective and responsive approaches to address complex challenges and create healthier, more resilient urban environments.

#### 3.1.9 A novel synthesized framework for data-driven environmental planning in sustainable smart cities

**Fig. 3:**
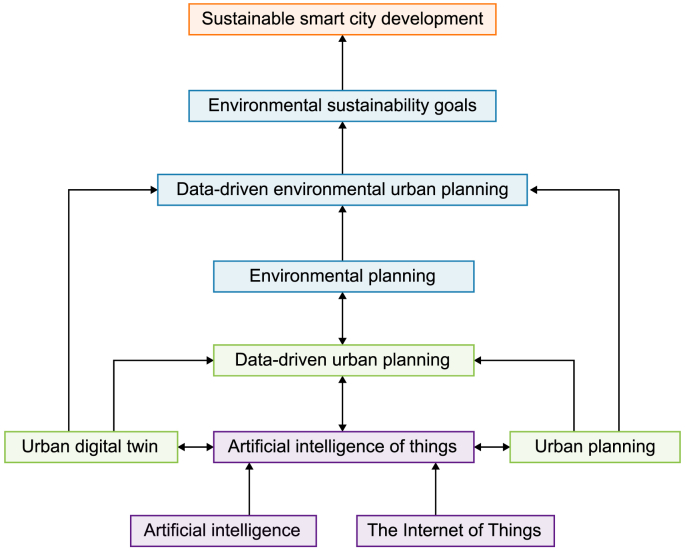
A novel synthesized framework for data-driven environmental planning in sustainable smart cities.

Derived from the analysis and synthesis of the theoretical and practical foundations driving the convergence of AI, AIoT, UDT, data-driven urban planning, and environmental sustainability in sustainable smart cities, the novel conceptual framework (Fig. 3) illustrates an interconnected ecosystem among these critical elements. This interconnectedness establishes a comprehensive framework for advancing sustainable smart cities. At its core, this framework integrates cutting-edge technologies to drive data-driven environmental urban planning practices. AI and AIoT empower UDT and facilities’ urban planning by providing sophisticated algorithms and real-time data processing capabilities, enabling predictive analytics, optimization, and decision-making in urban environments. This synergy fosters smarter and more efficient resource allocation, infrastructure management, environmental management, and service delivery, ultimately enhancing the sustainability of urban ecosystems.

Data-driven urban planning, facilitated by AIoT and UDT technologies, serves as the central element of this framework. It leverages vast amounts of urban data to inform planning decisions, infrastructure design, and policy formulation, leading to more resilient, adaptable, and environmentally conscious cities. In this context, it is shaped by environmental planning, which prioritizes sustainable development practices and aims to mitigate the adverse impacts of urbanization on the environment. Urban planners can anticipate future challenges, identify optimization opportunities, and develop strategies to mitigate environmental impacts by harnessing data analytics and predictive modeling.

Environmental sustainability is a central tenet of this framework, guiding the integration of AI, AIoT, UDT, and data-driven urban planning practices. Sustainable smart cities can implement proactive measures to address environmental concerns such as air and water pollution, energy consumption, waste management, and climate change mitigation by leveraging advanced technologies and data-driven insights. The framework emphasizes the importance of promoting strategies that support long-term environmental sustainability and resilience in urban areas. In essence, this conceptual framework represents a holistic approach to sustainable urban development, where AI, AIoT, UDT, data-driven urban planning, and environmental sustainability converge to create intelligent, livable, and environmentally friendly cities.

### 3.2 Adoption of artificial intelligence and artificial intelligence of things in data-driven environmental urban planning

The preceding subsection has offered foundational insights and synthesized perspectives on the substantial impact of prominent data-driven solutions and approaches on shaping the multifaceted landscape of urban planning. These insights and perspectives hold particular significance in sustainable smart cities, highlighting the crucial role of advanced technologies and environmental strategies in their evolution. This subsection particularly emphasizes the significance of AI and AIoT-based tools and methodologies in enhancing data-driven environmental urban planning processes and practices. It synthesizes research examining the intersection of AI, AIoT, and data-driven environmental urban planning, encompassing two sets of studies on their linkages: partial and comprehensive explorations.

#### 3.2.1 Research on partial explorations of linkages

The growing body of research on utilizing and integrating AI and AIoT in data-driven urban planning underscores their instrumental role in advancing environmental sustainability objectives. This has led to the emergence of novel approaches to data-driven environmental planning to tackle the mounting challenges of urbanization, ecological degradation, and climate change encountered by smart cities. Such developments reflect a response to the increasing scholarly interest in, and policy emphasis on, environmentally sustainable urban development practices, particularly in recent years (see Ref. [7] for a bibliometric analysis). They particularly highlight the crucial contributions of AI and AIoT in enhancing operational efficiency mechanisms and strategic planning methods [8,19,21,22,24,27,104,105]. In light of this, the conventional approaches to urban planning, which have long grappled with the dynamic properties and behaviors of complex systems [[78], [79], [80], [81]], have served as a fertile ground and significant opportunity for the integration of AI and AIoT to deepen our understanding of the complexities inherent in urban environments. In this context, any city or urban area that adopts AI will need to integrate it fully into the wider urban planning practice to handle urban complexities [106]. Nevertheless, bridging the gap between AI capabilities and planning applications for sustainable smart urban development is challenging. These challenges stem from the intricate and multidimensional nature of sustainable development [107,108], compounded by the realities of urban life (e.g., citizen/stakeholder behaviors, socioeconomic factors, political dynamics, and governance challenges) and the intricacies of human-environment interactions adds another layer of complexity [109].

In data-driven environmental planning, the symbiotic relationship between AI and IoT and the synergistic potential of AIoT offers unprecedented opportunities for enhancing decision-making processes and driving sustainable urban development initiatives forward. AIoT provides access to massive real-time data on urban systems and dynamics and applied intelligence capabilities, thereby improving various facets of data-driven environmental planning. Table 1 outlines the distinctive contributions of AI, IoT, and AIoT across various domains of sustainable smart cities in environmental sustainability and climate change. These insights are derived from a wide-ranging collection of relevant studies, encompassing both comprehensive explorations [[8], [9], [12], [13], [21], [22], [23], [66], [104], [110], [111], [112]] and more targeted investigations, both focused on the utilization of AI and AIoT to tackle complex environmental challenges.

**Table 1:** Integration of AI, IoT, and AIoT in data-driven environmental urban planning.

Area	AI contribution	IoT contribution	AIoT contribution
Sustainable transportation	Predictive modeling for traffic flow optimization, route planning, and infrastructure development.	Real-time monitoring of traffic patterns and transportation systems.	Integration of AI algorithms for traffic prediction with IoT sensors for real-time data collection and analysis.
Energy efficiency	Optimization of energy distribution and consumption.	Smart grid management for efficient energy usage.	AI-driven analysis of IoT data to identify energy-saving opportunities and optimize energy usage in real-time.
Water management	Prediction of water quality and quantity in distribution.	IoT sensors for monitoring water levels and quality.	AI algorithms analyzing IoT data for early detection of leaks and contamination and optimizing water distribution.
Waste management	Intelligent waste sorting and recycling.	IoT sensors for waste level monitoring and collection.	AI-powered optimization of waste collection routes based on IoT data, predictive maintenance for waste management systems, and waste composition analysis.
Environmental monitoring	Automated analysis of satellite imagery for environmental changes.	IoT devices for real-time data collection and monitoring of environmental factors.	Integration of AI for image recognition and pattern analysis with IoT devices for comprehensive environmental monitoring.
Climate change	Climate modeling and simulation for mitigation strategies.	IoT sensors for monitoring climate-related parameters and gathering climate data.	AI-enhanced analysis of IoT data for predicting climate trends and events, optimizing mitigation strategies, and implementing adaptive measures.
Land use	GIS data analysis for land use planning and management.	IoT devices for monitoring land use changes and soil conditions.	AI-driven optimization of land use planning, zoning optimization, and green space allocation through analysis of IoT data and predictive modeling.
Biodiversity	Species identification and habitat mapping.	IoT sensors for real-time wildlife tracking, habitat surveillance, and biodiversity indicators.	AI algorithms analyzing IoT data to identify biodiversity hotspots, track changes, and inform conservation efforts.
Ecosystem	Ecosystem modeling and simulation.	IoT devices for monitoring ecosystem health and dynamics.	AI-driven analysis of IoT data to assess ecosystem resilience, identify threats, and inform conservation strategies.

Targeted investigations pertain to renewable energies systems [10], hydrogen-based hybrid renewable energy systems [113], photovoltaic power generation forecasting [114], energy management systems [115], energy demand side [116], waste management [117,118], sustainable transportation development [119], ecosystem services [98], biodiversity protection [120], urban water resource management [99,121], air pollution [122], flood resistance [123], flood risk assessment [124], and flood prediction [16]. Important to note is that the energy sector is a primary user of AI in smart cities [8,125], with various technologies supporting the monitoring, analysis, and application of planning processes to combat pollution in urban environment. As noted by Sanchez [4], the prevailing discourse on AI in planning predominantly revolves around smart city technologies focused on data capture and analysis for optimization processes such as transportation and energy management. Conversely, relatively scant attention is given to AI's utilization in urban planning and decision-making endeavors, encompassing scenario planning and generative designs [4].

AIoT offers support across a spectrum of urban planning practices, facilitating the integration of sustainable urban technologies like IoT. Given the vast scale of IoT data, AI aids city development and regional development while considering environmental sustainability [126]. AI can assist in regional planning by predicting future growth and development [127], and the emerging applications of AIoT provide valuable data-driven insights for planning at both urban and regional levels [20,128]. By integrating AI and AIoT innovations into urban planning and design processes and frameworks, sustainable smart cities can achieve long-term environmental sustainability, enhance environmental quality, and ultimately improve the overall well-being of urban inhabitants.

These studies illustrate the role of AI and AIoT in shaping and supporting applications for planning the natural and physical environment. Their findings provide a comprehensive understanding of these advanced technologies' technical capacity and synergistic potential in fostering the evolution of sustainable smart cities towards greater efficiency, resilience, and livability. AI contributes by enabling data-driven insights and advanced computational and analytical capabilities, while IoT complements these efforts with real-time monitoring and data collection through a network of interconnected sensors and devices. AI algorithms, powered by vast datasets generated via IoT infrastructure, enable real-time analysis and informed decision-making for data-driven environmental planning. This technological convergence provides urban planners with tools to model and simulate various scenarios, predict environmental impacts, identify intervention measures, and devise strategies for more efficient land use, resource utilization, ecological resilience, and infrastructure design.

Data-driven environmental planning is evolving into a symbiotic relationship with AI and AIoT, providing urban planners with enhanced capabilities to seamlessly integrate urban systems, coordinate urban domains, and couple urban networks. This synergistic integration empowers them to conceive and implement integrated solutions for tackling complex environmental challenges. It creates a dynamic system where advanced technologies contribute to adaptive and proactive urban environments. This innovative approach aligns with the goals of sustainable smart cities, fostering a harmonious balance between urban development, technological innovation, and environmental preservation.

#### 3.2.2 Research on comprehensive explorations of linkages: machine learning, deep learning, computer vision, and natural language processing

Research systematically or specifically investigating AI or AIoT and data-driven urban planning linkages has gained prominence recently. Scholars have investigated the intricate interplay between these domains, aiming to uncover their potential synergies and implications for sustainable urban development. Recent publications indicate a surge in the utilization of AI models, especially ML, DL, CV, and NLP, as a pivotal tool for decision support in data-driven urban planning. Kamrowska-Załuska [92] evaluates the potential use of AI-driven urban big data analytics to support city design and planning. The author presents a conceptual framework to examine how these tools influence urban design in the context of urban change. The discussion covers the implications of applying AI-based tools and geo-localized big data in addressing specific research problems in urban planning and design and their impact on planning practices. The insights and recommendations are tailored for urban planners intrigued by the emerging field of AI-based urban big data analytics and urban theorists seeking innovative approaches to understanding urban change.

Koumetio Tekouabou et al. [2] conducted a comprehensive analysis of AI-based methods for smart and sustainable urban planning, identifying common urban planning issues and the predominant data and ML and DL techniques employed. By synthesizing the existing literature, the authors identified key areas of focus, data sources, and geographic regions, offering insights for urban planning researchers and practitioners. They also discussed prevailing trends, critical issues, current challenges, existing gaps, and future research directions in this dynamic field. They reveal that DL methods are the preferred choice for addressing land use, buildings, and climate challenges, predominantly relying on satellite image data. Similarly, Son et al. [5] systematically explored data-driven urban planning for smart and sustainable development, focusing on applying AI technologies in urban contexts. The authors highlighted the role of AI, especially ML and DL, in addressing economic, social, and environmental challenges in cities while emphasizing the importance of collaboration and data-driven approaches. The findings underscore the need for integrating AI into urban planning processes to achieve smarter and more sustainable cities.

The advancements in CV methods and their robust computing capabilities have shown promising opportunities for advancing sustainable urban development by enhancing data-driven planning practices. To gain a better understanding of how CV can be integrated into the urban planning process to create more sustainable smart cities, Marasinghe et al. [3] conducted a systematic review of CV applications in urban planning, highlighting the opportunities offered by CV to support various planning tasks while acknowledging existing challenges. Their findings unveil several key points: (a) CV has the potential to facilitate numerous urban planning tasks, spanning from data collection and analysis to issue identification and prioritization, public participation, plan design and adoption, and implementation and evaluation; (b) CV can enhance decision-making processes through diverse visual information, though its limitations must be taken into account; and (c) CV utilization in urban planning holds promise for advancing efforts in sustainable urban development.

In addition, Koumetio Tekouabou et al. [2] demonstrated that DL and CV can identify global urban issues related to land use, buildings, climate, and the natural environment. CV methods in urban sensing can collect various environmental and socioeconomic data from vast collections of images and videos [129]. Understanding visual environments entails more than just identifying physical objects and environmental attributes; it also encompasses human experiences and perceptions [130]. Song et al. [5] emphasized the importance of CV as a key element in advancing AI for enhancing urban planning and design by investigating user experiences. The authors highlight the significance of this user knowledge in providing researchers and professionals with empirical evidence to evaluate planning and design interventions. CV enables professionals to analyze diverse locations, leveraging fine-grained information on microscale urban features and large datasets, thus enabling them to gain insights and predict people's perceptions of urban environments [131].

Additionally, applications of ML for CV tasks in the domain of urban planning and design include exploring associations between people's urban density and urban characteristics [132] and analyzing associations between eye-level urban design quality and on-street crime density [133]. Urban planners and policymakers can gain valuable insights and empirical evidence to guide decisions in sustainable urban planning and design by utilizing CV techniques to investigate and assess the spatial correlation and underlying factors contributing to diverse urban challenges and prospects [3]. The emergence of spatial-explicit geospatial AI (GeoAI), which integrates spatial attributes and reasoning into AI computations, is increasingly prevalent in urban studies [134,135], offering diverse applications and opportunities for environmentally sustainable urban planning and development.

In data-driven urban planning, CV techniques have been utilized for different planning and design activities. These tasks include evaluating planning outcomes, monitoring designs, assessing spatial vitality and user experiences, and enhancing planning and design processes. AI has enabled urban researchers, designers, and planners with extensive proficiency in utilizing CV for designing, observing, and modeling urban environments and planning, evaluation processes, and stakeholder participation (Wael et al., 2022). Visual data are of critical importance in the process of urban planning and design, thereby the significance of CV [130,136] as a valuable tool for decision-making and planning processes [136]. In particular, CV holds significant promise in its ability to provide accessible and cost-effective tools for urban assessment and modeling [137,138].

Furthermore, CV plays an important role in the sensor city approach, which employs IoT technologies to gather and synthesize data for enhancing urban planning and sustainability efforts [139]. CV can empower urban planners and policymakers to evaluate diverse scenarios and their impacts on the sustainability and resilience of cities throughout the planning cycle and to track the progress and outcomes of their interventions [138,140]. Overall, CV has emerged as a pivotal technology for smart and sustainable urbanism initiatives, which strive to create resilient and environmentally friendly urban environments through data-driven methodologies that enhance sustainable urban planning [141,142].

Urban planners have traditionally grappled with copious textual content encompassing plans, policies, reports, stakeholder engagement, and community feedback from public engagement initiatives. NLP emerges as a vital tool for analyzing such textual data, enabling the extraction of valuable insights (e.g., sentiment analysis, topic modeling, and named entity recognition) to understand public opinions, identify trends, and inform decision-making processes in urban planning and design. Planning scholars have leveraged NLP techniques to analyze social media content, capturing public sentiment and opinions on various subjects across diverse temporal and spatial contexts [143]. Moreover, NLP facilitates the examination of perceptions regarding urban parks based on environmental features [144], the analysis and visualization of users' sentiment towards the built environment [145], the extraction of relevant information from planning documents [146,147], and the integration of spatial development plans [148]. Kaklauskas et al. [149] developed an “affective system” for researching emotions in public spaces for urban planning, drawing from behavioral economics, the psychology of judgment and decision-making, and human emotional affective and physiological states. This framework enhances urban planners' ability to analyze planning processes effectively and make informed decisions. Considering the above insights, integrating NLP techniques into data-driven urban planning offers a valuable avenue for enhancing environmental sustainability. By analyzing diverse textual data, planners can identify emerging environmental trends, understand public sentiments regarding environmental issues, and utilize these insights to inform decision-making processes to foster sustainable urban development initiatives.

Worth noting is that in data-driven environmental planning, ML, DL, CV, and NLP play interconnected roles in harnessing data for informed decision-making. ML algorithms analyze historical and real-time environmental data to identify patterns and trends, while DL models process complex environmental datasets with multiple layers of abstraction. To assess environmental conditions and changes, CV techniques extract spatial information from imagery and sensor data. NLP enables the extraction of insights from textual data, including reports, policies, and public feedback. Sustainable urban development is further enhanced by the integration of these technologies. Together, these empower planners to understand and model urban systems and dynamics and address environmental challenges effectively in urban areas [[5], [6]].

However, the adoption of AI and its subset models in urban planning is anticipated to be a gradual process, demanding considerable time and resources [4] — despite the promising prospects and opportunities it holds, together with AIoT, for advancing environmental goals in sustainable smart cities. Despite the increasing research on AI-driven urban planning, challenges persist in translating research into practical applications, primarily due to the concerns expressed by urban planners about adopting AI in the field [3,4,150]. Nonetheless, given the anticipated pivotal role of AI and AIoT in strategic city planning aimed at bolstering environmental sustainability and resilience [[2], [5],^19^,^92^], planners must proactively prepare for the transformative implications this herald for sustainable urban development. This includes reimagining how future cities will be planned, designed, and managed. To prepare planners for the AI revolution, Sanchez [4] outlined several challenges that must be addressed, including.

• Overcoming fear and uncertainty surrounding AI adoption.
• Acquiring new skills necessary for leveraging AI technologies effectively.
• Adapting to changing data needs and considerations inherent in AI-driven planning processes.
• Establishing clear goals to guide AI implementation strategies.
• Ensuring transparency and explainability in AI systems.
• Mitigating bias encountered in AI algorithms and decision-making processes.
• Addressing ethical concerns arising from using new methods and data in AI-driven planning endeavors.

Sanchez et al. [6] underscored the significance of integrating AI-related techniques into urban planning, noting the timeliness of this endeavor in light of the growing availability of data, faster processing speeds, and the development of planning-related applications. However, urban planners need to ensure the effective integration of AI and AIoT technologies and their responsible use in future urban planning endeavors. Andrews et al. [1] investigated the expanding role of AI in the planning profession and its potential implications for communities. The authors offered insights into how planners can effectively integrate AI into their practices while ensuring fairness and inclusivity.

### 3.3 Artificial intelligence, artificial intelligence of things, and urban digital twin: advancing data-driven environmental urban planning

This subsection explores the profound impact of AI- and AIoT-driven UDT on data-driven environmental planning in sustainable smart cities. It highlights the effectiveness of this technological integration in shaping and enhancing sustainable urban development practices, particularly in simulating various scenarios, predicting outcomes, and optimizing resource management strategies. By providing insights into the evolving landscape of AI and AIoT adoption in data-driven urban planning systems through UDT, it underscores their significant role in advancing environmental sustainability goals.

#### 3.3.1 Applications of urban digital twin for environmentally sustainable urban development and planning

Integrating AI and AIoT with UDT in city modeling and simulation has yielded positive outcomes in advancing data-driven environmental planning and the design of sustainable smart cities. Several recent studies have explored this synergistic approach's potential, uncovering valuable insights and applications. Table 2 summarizes relevant studies discussing the application of both AI/AIoT in UDT environments. It illustrates how AI/AIoT UDT are integrated to enhance planning, infrastructure management, energy systems, building design, transportation systems, flood risk assessment and prediction, urban dynamics, and more.

**Table 2:** Integrating AI and AIoT with UDT for environmentally sustainable smart city applications.

Citations	AI techniques	UDT type	UDT simulation focus	Key contributions
Austin et al. [57]	Semantic knowledge representation, ML	Smart city	Energy usage analysis in buildings	Proposal of a smart city DT architecture for complementary roles in data collection, event identification, and automated decision-making.
Shen et al. [34]	ML, AI	Positive energy districts (PEDs)	Optimization of PEDs	Optimization of livability in urban environments for sustainability dimensions.
Lv et al. [151]	DL, convolutional neural network (CNN), support vector regression (SVR)	Cooperative intelligent transportation system (CITS)	Security and performance analysis, path planning optimization, impact analysis of transportation network	Improved DL algorithm for CITS DTs and enhanced security and performance.
Bindajam et al. [152]	SVM, artificial neural network (ANN), cellular automata (CA)	Land use and land cover (LULC)	Past-to-future LULC analysis, ecosystem services estimation, sensitivity analysis	Prediction and analysis of LULC dynamics and impact on ecosystem services.
Agostinelli et al. [32]	AI, ML	Building energy management	Energy efficiency, renewable energy production	Assessment of different scenarios for energy efficiency interventions and renewable energy production in a residential complex and evaluation of the effectiveness of integrative systems for renewable energy production.
Lu et al. [153]	AI, federated learning	Smart city with multi-energy system	Smart transportation, smart energy grid	Proposal of a DT-based smart city system to address operational challenges in complex environments.
Fan et al. [154]	AI, multi-actor game-theoretic decision making, dynamic network analysis	Disaster city	Situation assessment, decision making, coordination	Vision for leveraging AI and human intelligence in disaster management through a DT paradigm
Deena et al. [155]	AI, ML	Building energy management	Testing energy-efficient intervention scenarios and optimizing energy usage	Development of a DT-based optimization system for energy management in a residential area
Strielkowski et al. [156]	AI	Energy sector	Safety, security, and reliability of energy networks	Overview of DT in smart grids and its application in improving energy network safety and reliability, as well as use cases for design, operation, control, and maintenance planning in energy utilities.
Wu et al. [35]	AI, classificaton algorithms	Transportation	Transportation infrastructure	Classification and prediction of the intelligent development of transportation infrastructure based on space type, function type, and facility use.
Almusaed and Yitmen [33]	AI simulation models	Smart building design	User experience optimization, building performance prediction, intelligent design feature creation	Integration of AI simulation models with DT and development of intelligent building design features.
Ye et al. [36]	Multi-agent interactions, clustering algorithms	Infrastructure resilience	Conceptual framework development	Integration of AI into UDT, development of human-centered UDT for community resilience.
Liu et al. [135]	Genetic and evolutionary AI algorithms	Freight parking management	Real-time parking connectivity, logistics efficiency optimization, urban resource allocation	Integration of cognitive DTs with logistics, improved logistics efficiency, and resource allocation optimization.
Manocha et al. [157]	Fuzzy logic (FL)	Flood prediction	Flood forecasting, situational analysis, blockchain security	DT-based flood prediction, improved decision-making for flood management, and enhanced data security.
Nica et al. [158]	Predictive modeling algorithms, DL	Cities	Sustainable urban governance, smart city environments	Configuration of immersive virtual spaces through various technologies in DT cities and optimization of IoT-based smart city environments through DT simulation, DL sensing technologies, and urban data fusion.
Sabri et al. [159]	Geosimulation, spatial-visual intelligence, GeoAI	Spatially-explicit	Smart water infrastructure and flood management	Incorporation of accurate location-based data using GIScience methods and roadmap for creating spatially-explicit UDT for smart urban water and flood management systems.
Salunke A. A [160].	Reinforcement learning (RL)	Traffic flow	Urban dynamics, traffic flow optimization	Introduced RL empowered DT in transportation, energy, and planning applications.
Gkontzis et al. [56]	AI-driven data analyses, forecasts	Neighborhood level	Enhancing urban resilience	Enhanced urban resilience; visualization, analysis, and prediction of urban system responses; and improvements in urban functionality, resilience, and resident quality of life.
Ziakkas et al. [161]	ML, AI	Advanced air mobility (AAM) systems	Certification of AAM systems	Certification of advanced AAM systems, design and remote testing of eVTOL aircraft simulator prototypes, and effective and efficient AAM design while mitigating AI-related risks.
Kamal et al. [162]	Deep reinforcement learning (DRL)	Traffic signal	Traffic signal control for reduced CO_2_ emissions and fuel consumption	Proposed a DT-based adaptive traffic signal control approach for reduced CO_2_ emissions and fuel consumption.

The coupling of UDT's detailed replica with AI's analytical prowess allows for more accurate forecasting of urban and environmental conditions. Researchers have successfully simulated and predicted the impact of various urban development scenarios related to different spheres of urban life, contributing to proactive data-driven environmental urban planning. By leveraging AI algorithms in UDT, cities can efficiently monitor and allocate resources, such as energy, water, and waste [33]. Shen, Saini, and Zhang [34] focused on PEDs in terms of integrating various systems and infrastructures to facilitate optimal interactions among buildings, mobility, energy, and advanced technologies to enhance environmental sustainability. This exploration focuses on both the process of creating a DT for PEDs and its optimization for enhancing livability. Moreover, the combination of AI and UDT has proven particularly effective in addressing urban mobility and transportation challenges. Through sophisticated simulations, AI-driven UDT models can predict traffic patterns, optimize public transportation routes, and propose intelligent traffic management strategies [35,[160], [162]]. This contributes to reduced congestion and improved transportation efficiency and aligns with the broader goal of sustainable urban mobility in terms of CO_2_ emissions reduction. Moreover, AI-driven UDT applications have demonstrated their capacity to support informed decision-making in land use planning [152], central to environmental sustainability regarding ecosystem services, biodiversity conservation, urban heat island mitigation, and water resource management. For example, AI has been used in urban planning research to measure the urban heat island effects [163].

The findings of these studies underscore the potential of integrating AI within UDT frameworks to support holistic approaches to data-driven environmental planning. This integration ensures the conservation of natural resources, the enhancement of ecosystem services, and the reduction of environmental impacts, all contributing to the overarching goal of fostering environmental sustainability in urban development. Furthermore, the insights derived from integrating AI with UDT for city modeling and simulation in sustainable smart cities mark significant progress in various facets. Advanced simulation models empower decision-makers to pinpoint areas for improvement, implement targeted interventions, and elevate the overall sustainability performance of urban environments.

**Fig. 4:**
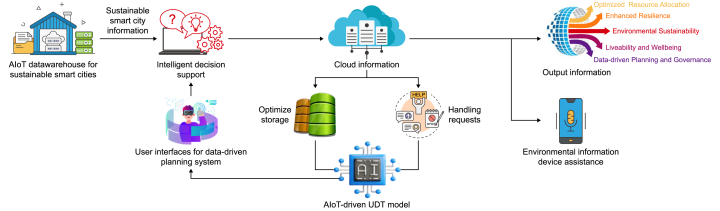
An AIoT-driven UDT framework for data-driven environmental planning in sustainable smart cities.

Based on the insights gathered from Table 2, Fig. 4 illustrates an AIoT-driven UDT framework designed to enhance data-driven environmental planning in sustainable smart cities. This framework incorporates various components, including an AIoT data warehouse, AIoT-driven UDT models, environmental information device assistance, cloud interfaces, and decision support systems. By integrating these elements, the framework addresses the needs of a sustainable smart city ecosystem, enabling the generation of AIoT-driven UDT insights essential for effective data-driven environmental planning initiatives.

#### 3.3.2 The dynamic interplay of urban digital twin, artificial intelligence, and artificial intelligence of things in data-driven urban planning and design

This subsection explores the dynamic interplay of UDT, AI, and AIoT in data-driven urban planning and design, providing insights into their synergistic and collaborative capabilities for advancing sustainable and intelligent urban environments. It emphasizes the critical role of AI- and AIoT-driven UDT approaches in shaping the trajectory towards environmentally sustainable smart urban futures.

Beckett [29] explored the integration of UDT, 3D modeling, visualization tools, and spatial cognition algorithms in the context of AI-based smart city design and planning, providing a nuanced understanding of the evolving landscape of urban development. The author accentuated the pivotal role of AI in UDT, demonstrating how its integration enhances urban design and planning strategies. As evidence of 3D virtual simulation technology, data-driven urban analytics, and real-time decision support systems continues to grow, it has become crucial to explore the integration of IoT-based DT with AI-based urban design and planning, along with real-time urban data. This will propel urban planning and design processes to new heights of sophistication. The synthesis of prior findings in this research offers insights into the evolving landscape of data-driven planning and design technologies, shedding light on the effective utilization of remote multi-sensing data, simulation modeling algorithms, and augmented analytics tools in pursuing sustainable and intelligent urban development. Lastly, while not explicitly focused on environmental sustainability aspects, the implications of this research suggest the potential contribution of these integrated approaches to data-driven environmental planning and design, thereby advancing smart, sustainable urban development initiatives.

The DT algorithms proposed by Zvarikova et al. [164] constitute an approach that leverages 3D spatiotemporal simulations in virtual urban environments. The authors deploy diverse urban sensing data to drive ML, CNNs, and advanced DT algorithms, creating a robust and sophisticated framework for urban simulation. This enhances the accuracy of spatiotemporal modeling and highlights the potential for AI, particularly ML, and DL, to transform the way virtual urban environments are simulated. Furthermore, using these AI algorithms holds promise for enhancing data-driven environmental planning processes through more accurate and comprehensive urban simulations, facilitating informed decision-making for sustainable urban development.

Similarly, the UDT architecture introduced by Austin et al. [57] highlights the importance of semantic knowledge representation and reasoning in conjunction with ML, providing a valuable contribution to the intersection of AI and urban planning. This integration enhances data collection, event identification, and automated decision-making. The emphasis on ML techniques within the UDT architecture signifies a notable advancement in applying AI and ML in urban contexts. The study's focus on energy usage analysis in buildings in the Chicago Metropolitan Area exemplifies the practical impact of the successful integration of semantic and ML approaches, demonstrating the potential of AI and ML to drive intelligent decision-making processes in the context of smart cities. The findings emphasize AI and ML's innovative role in shaping urban planning's future, with implications for sustainable smart city development.

In addition, Zayed et al. [30] addressed the promising frontier for DT applications across various real-world domains driven by the convergence of AI and IoT. The authors comprehensively explored the burgeoning integration of DT technology with AI techniques and IoT applications. They focused on the challenges, opportunities, and recent developments in this innovative domain. They further examined the incorporation of AI and DT in developing IoT-based applications, present tools for implementing DT systems, and analyze recent AI approaches in the DT domain. The study contributes to understanding AI-driven DT applications and identifies open research directions in this evolving field. Wang et al. [165] examined the Internet of digital twins (IoDT), emphasizing its potential for facilitating dynamic data exchange and mission cooperation across physical and virtual entities. IoDT refers to a network of interconnected digital replicas of physical entities, enabling data exchange and collaboration across various domains. The authors provided a detailed exploration of IoDT, including its architecture and enabling technologies. They argued that IoDT, despite its promise, faces significant challenges due to its decentralized nature and information-centric routing.

Though not directly addressing the concept, Zayed et al. [30] and Wang et al. [165] both hint at the evolving landscape of artificial intelligence of digital twin (AIoDT). This concept, introduced in Ref. [19,166], emphasizes the integration of AI with IoDT to bolster the functionalities and capabilities of diverse AIoT applications for environmentally sustainable smart cities. However, while Zayed et al. [30] focused on the integration of AI techniques with UDT technology in IoT applications, Wang et al. [165] explored the broader concept of IoDT. Despite their distinct scopes, the two studies revolve around the synergistic potential of AIoDT in facilitating dynamic data exchange, mission cooperation, and innovative sustainability solutions. By integrating the findings of these studies, a more nuanced understanding of AIoDT emerges, emphasizing its role in driving sustainable urban development and addressing critical challenges in data processing and exchange. Moreover, these studies collectively identify key research directions and pave the way for future advancements in AIoDT, positioning it as a cornerstone in the quest for innovative and resilient sustainable smart city solutions. In addition to enhancing functionality and capabilities for various real-world AIoT applications, the integration of AI with IoDT — AIoDT — holds significant promise for optimizing data-driven environmental urban planning processes. By harnessing AIoDT technologies, urban planners can gain valuable insights into environmental factors and develop more effective strategies for sustainable urban development.

Overall, the reviewed studies illuminate the groundbreaking potential of AI and AIoT in shaping the future of urban planning and design through UDT across theoretical, technical, and practical dimensions. By integrating ML, DL, CV, and NLP with UDT frameworks, urban scholars and planners are set to transform how sustainable smart cities can be planned and designed to realize environmental goals. These findings underscore the importance of leveraging AI- and AIoT-driven UDT systems to address the challenges of rapid urbanization and escalating ecological degradation, thereby fostering the development of more resilient, efficient, and livable urban environments.

### 3.4 Challenges, barriers, risks, and related mitigation strategies

This section examines the intricate spectrum of difficulties and solutions stemming from the convergence of AI, AIoT, UDT, data-driven urban planning, and environmental sustainability in sustainable smart cities, particularly emphasizing their practical applications. The main objective is to provide readers with a comprehensive understanding of the underlying dynamics of technological convergence and synergistic integration of these technologies, models, and domains in sustainable smart urban development.

#### 3.4.1 Data privacy and security concerns

In sustainable smart cities, where data integration and interconnected devices are pervasive, prioritizing the security and privacy of their infrastructures and systems is paramount. Implementing strong security and privacy measures is essential to guard against cyber threats and uphold the confidentiality of citizens' data. The rising utilization of AI and AIoT technologies, especially concerning integrating UDT frameworks in sustainable smart cities, signifies a crucial yet complex stage in urban development. As these technologies become integral to the urban fabric, concerns about the privacy and security of citizen’ data have gained considerable prominence and are impending the implementation of AI, AIoT, and UDT [[8], [28], [73], [167], [168], [169]] or AI/AIoT-driven UDT in various domains. Wang et al. [165] offered a comprehensive examination of IoDT architecture, security, and privacy, which inherently encompasses IoT — and extends to AIoT. The authors argued that security and privacy concerns hinder the deployment of IoDT (and hence AIoDT) due to inherent vulnerabilities and decentralized structure. They also presented state-of-the-art defense mechanisms in the context of IoDT. In light of the importance of security and privacy issues addressed in Wang et al.'s review [165] of IoDT, its implications extend to the broader domain of UDT, which is integral to data-driven environmental planning in sustainable smart cities. By identifying and addressing security flaws and privacy invasions in IoDT (and hence AIoDT), this work contributes to building a more robust foundation for implementing UDT. As UDT systems play a crucial role in monitoring and managing various environmental parameters, ensuring their security and privacy enhances their effectiveness in supporting data-driven environmental planning initiatives. Therefore, Wang et al.‘s study [165] indirectly supports the advancement of environmental planning practices by fortifying the underlying digital infrastructure essential for sustainable urban development.

The vast amounts of data collected through various sensors, devices, and interconnected systems pose potential risks to citizens, as personal information is increasingly susceptible to misuse and unauthorized access. Striking a delicate balance between harnessing the power of data for informed urban planning decisions through UDT and ensuring the protection of individuals' privacy rights has become an imperative task for city planners, policymakers, and technology developers alike [[28], [55], [169], [170]]. The complexity of UDT and the abundance of available data amplify data privacy threats, making it nearly impractical to ensure privacy by design principles. It is impossible to ensure that their integration maintains user anonymity [171]. The real challenge lies in navigating the fine line between leveraging data to enhance the efficiency, functionality, sustainability, and livability of urban environments and addressing the valid concerns of citizens regarding the privacy and security of their personal information. Implementing UDT involves creating detailed virtual replicas of urban environments’ physical and functional aspects, necessitating the integration of diverse datasets from multiple sources. The multifaceted nature of these data, encompassing everything from energy consumption patterns to travel behavior and mobility mode and waste management, amplifies the complexity of the privacy and security landscape.

In a similar vein, the security and privacy concerns associated with AI and AIoT have been extensively separately discussed, reflecting the critical importance of safeguarding personal data and ensuring ethical and responsible use of AI and IoT technologies [8,[73], [167]]. Urban planners, technology developers, and policymakers must collaboratively devise robust frameworks that incorporate privacy-enhancing mechanisms, stringent data protection measures, and transparent governance structures to instill confidence among citizens while realizing the transformative potential of AI and AIoT technologies in shaping the future of sustainable smart cities. Recommendations include transparent practices, accountability, transparency, bias mitigation, data minimization, informed consent, and ethical design. Policymakers are urged to develop adaptable regulations, prioritize privacy and security, and involve stakeholders. However, the research emphasizes both the imperatives and challenges associated with regulatory compliance and governance frameworks [[170], [172]], including the legal and licensing complexities arising from UDT with partially open data [173]. Ensuring data privacy and security while collecting and sharing sensitive information in UDT poses ethical and regulatory challenges.

#### 3.4.2 Ethical and social considerations

Integrating AI, AIoT, and UDT marks a transformative leap in modeling, simulation, and data-driven decision-making capabilities. However, this newfound power necessitates carefully examining ethical and social dimensions apart from privacy and security. Ethical and social considerations entail fairness, bias, transparency, and accountability in decision-making processes [8,[169], [174], [175], [176]], with far-reaching implications. The dynamic interplay of AI, AIoT, and UDT requires a nuanced ethical and social approach, recognizing its potential implications for various aspects of urban life, including management, planning, governance, and services in the context of sustainable smart cities. A primary challenge is ensuring fairness in AI algorithms, particularly when monitoring, modeling, and simulating urban environments and processes within UDT frameworks. Algorithmic bias has the potential to perpetuate and exacerbate existing disparities in sustainable smart cities, leading to unequal representation and outcomes [177]. Fairness, extensively discussed in the broader AI field [175,178] and AIoT domain [8,167], becomes crucial in the UDT context. Obstacles to achieving transparency include the complexity of UDT models [25] and reluctance towards sharing data [26,179]. Research has demonstrated that a lack of transparency diminishes citizens' participation in UDT [171].

Responsible and transparent use of AI and AIoT in the development and implementation of UDT is imperative to enable urban planners and policymakers to comprehend decision-making processes and identify and mitigate potential areas of bias. While numerous scholars contend that the design of UDT frequently overlooks social and ethical considerations [53,59,179,180], future research on data fairness and transparency is essential to enhance public acceptance and citizen inclusion in UDT initiatives in sustainable smart cities [28]. Moreover, accountability mechanisms must be established to trace decisions made by AI algorithms back to their sources, ensuring that responsible parties can be answerable for the consequences of AI- and AIoT-driven decisions and actions within the UDT framework. Challenges such as legal uncertainties, unclear responsibilities for accountability [55], and conflicting goals among stakeholders [181] may lead to resistance from city officials or residents to the implementation of UDT [182]. Addressing these concerns is vital to fostering trust and acceptance in adopting UDT for sustainable smart cities.

As UDT become increasingly embedded in urban planning processes, ethical and social considerations should not be treated as an afterthought but as integral components in designing and adopting AI and AIoT technologies. Collaborative efforts among technologists, urban planners, ethicists, and policymakers are essential to establish robust ethical and social frameworks that guide the responsible deployment of AI and AIoT-driven UDT. In particular, Andrews et al. [1] stress planners need to comprehend AI's ramifications to harness its benefits judiciously and address potential risks, such as exacerbating societal disparities. The authors propose actionable strategies for planners to embrace AI responsibly, enhance planning procedures, and prioritize human-centered approaches. Additionally, they advocate for ethical AI practices and underscore planners' need to actively shape AI-based planning tools to foster equitable and inclusive outcomes for communities. Addressing ethical and social challenges is crucial for sustainable smart cities to fully leverage the potential of integrating AI and AIoT with UDT while upholding fairness, equality, transparency, and accountability principles. This proactive approach ensures that technological advancements align with ethical and social considerations, fostering a harmonious coexistence between technological innovation and societal well-being in sustainable urban development.

#### 3.4.3 Explainable artificial intelligence and interoperable machine learning

XAI and IML intersect with ethical and social issues in UDT by promoting transparency, accountability, and fairness in decision-making processes. They address bias, privacy, and trust concerns, ensuring that AI/AIoT-driven UDT systems are understandable and interpretable for various stakeholders. Explainable AI (XAI) and interpretable ML (IML) encounter significant challenges in AI, AIoT, and, consequently, AI/AIoT-driven UDT solutions for sustainable smart cities. They strive to enhance AI models' clarity, comprehensibility, and credibility for stakeholders engaged in sustainable urban development. While XAI elucidates the decision-making process of AI systems, IML focuses on creating ML models that yield easily interpretable outcomes. XAI and IML are essential for developing AI, AIoT, and associated UDT systems that foster trustworthiness and effective human-AI interaction, which are crucial for informed decision-making in sustainable smart cities. However, numerous challenges arise in this context, including complexity and interpretability, black-box models, algorithmic bias, the trade-off between accuracy and interpretability, data privacy and security, and dynamic urban environments [176,[183], [184], [185], [186], [187]]. The primary challenge lies in empowering AI/AIoT-driven UDT systems with significant capabilities while simultaneously ensuring they can explain intricate decision-making processes to various domain experts.

Recent research has concentrated on XAI to address concerns regarding the application of AI across various domains. For example, Mayuri, Vasile, and Indranath [187] explored the application of XAI/IML techniques to render AI/ML models explainable/interpretable. Ghonge [183] discussed several XAI case studies, use cases, and their impacts and challenges in smart city applications. Javid et al. [184] offered a comprehensive examination of XAI in smart cities, delineating current and future developments, trends, enabling factors, use cases, challenges, and solutions. Among particular relevance to this study, Jagatheesaperumal et al. [188] explored the transformative impact of XAI on the AI landscape, focusing specifically on its role in bolstering trust among end-users regarding machine interactions. Indeed, in light of the exponential growth in IoT interconnected devices, the need to ensure trustworthiness becomes increasingly paramount. The authors examined XAI frameworks, shedding light on their defining characteristics and relevance in the IoT context. They also provide insights into various XAI services commonly applied in IoT applications, including those pertinent to the Internet of City Things (IoCT). Additionally, they suggest implementation choices of XAI models for IoT systems in these applications. Their study is one of the first comprehensive compilations of XAI-based frameworks tailored to meet the evolving demands of future IoT use cases. Collectively, these endeavors highlight the critical role of XAI and IML in navigating the complexities of AI/AIoT-driven UDT solutions for sustainable smart cities.

#### 3.4.4 Interoperability and standardization

The seamless integration of diverse technologies in the complex web of the sustainable smart city ecosystem presents a complex puzzle — with challenges regarding interoperability and the lack of standardized protocols and frameworks. As sustainable smart cities evolve into more interconnected hubs of innovation, driven by the rapid progress of AI and AIoT technologies, integrating UDT into these cities' management and planning processes becomes increasingly pivotal for holistic urban development. However, a notable obstacle arises from the inconsistency among various technological components. The absence of interoperability and standardized practices hinders the smooth exchange of data and information between diverse systems. Consequently, this impedes the optimal functionality and broader adoption of UDT [28,[30], [169]]. Extensive discussions in the literature highlight the complexities surrounding interoperability and standardization, expressing concern about the lack thereof in prevailing UDT systems and infrastructures [[28], [169], [189], [190]]. These challenges are exacerbated by the inherent heterogeneity of urban environments, which necessitates adaptable and scalable solutions for data collection, processing, and analysis. Overall, the interoperability of systems and standards across different technological platforms may present technical hurdles in seamlessly integrating AI, AIoT, and UDT solutions.

UDT can be seen as an encapsulation of all the pertinent data applicable to the environmental planning of sustainable smart cities. Interoperability, the capacity of devices, systems, and processes to exchange contextualized information, emerges as a significant challenge for implementing UDT in sustainable smart cities. This capability allows UDT to act on shared data and information, enhancing planning decisions and predictive outcomes. Interoperability issues specifically arise when the diverse components of UDT, such as IoT devices, sensors, data platforms, analytical tools, and computing models, need to collaborate cohesively to support the comprehensive modeling and simulation capabilities of UDT [191]. The diverse origins and functionalities of these technologies and tools often result in proprietary systems that operate in silos, creating barriers to the smooth flow of information [170]. The data can be tightly coupled to applications, potentially losing information at the software interface [63]. This fragmented landscape impedes the real-time synchronization required for accurate 3D representations and undermines the collaborative potential of interconnected systems in sustainable smart cities. It is widely recognized that interoperability is one of UDT's most pressing unresolved issues [[55], [169]]. Interoperability and standardization in the urban context face various impediments, such as data silos created by infrastructure providers [192], organizational structures in government agencies [179], and the integration of multi-scale and multi-domain workflows [193,194]. Presently, data sharing between UDT and urban systems is limited, potentially hindering sustainable smart cities' progress in adopting UDT for advancing environmental planning [28].

To effectively tackle the challenges posed by the complex landscape of sustainable smart city infrastructure, it is imperative to prioritize developing and adopting standardized protocols. These protocols are pivotal in facilitating seamless communication and interoperability across sustainable smart cities' diverse technologies and systems [28,195]. Furthermore, addressing these challenges extends beyond technological harmonization; it highlights the critical need to establish interoperable interfaces cutting across various fields [172]. By fostering compatibility and synergy among disparate systems, the goal is to create a unified ecosystem that transcends individual technological silos. The crux of this endeavor lies in establishing a common means of data exchange and interaction. This foundational step is essential for unlocking the true transformative potential of UDT. Sustainable smart cities can harness the full power of AI and AIoT technologies through interoperability. This, in turn, facilitates more effective environmental planning initiatives, aligning with the broader objective of building cities that are not only technologically advanced but also resilient and environmentally conscious.

#### 3.4.5 Environmental risks

The rapid advancement and convergence of AI, AIoT, and UDT have ushered in a new era of technological innovation with transformative implications for sustainable urban development. While these technologies promise enhanced efficiency, connectivity, data-driven insights, and intelligent decision-making in urban planning, it is crucial to critically examine their environmental costs and risks. As sustainable smart cities embrace innovative solutions for advancing environmental sustainability goals, understanding the potential ecological impacts of AI, AIoT, and UDT integration becomes paramount. Table 3 presents the environmental challenges stemming from the widespread adoption of these technologies, alongside potential strategies to alleviate them. These insights are distilled from several studies on UDT (e.g., Ref. [[28], [55],[194], [196], [197], [198]]) and AI and AIoT (e.g., Ref. [7,[8], [40],^174^,[199], [200], [201], [202]]).

**Table 3:** Key environmental risks and mitigation strategies.

Environmental risks	Mitigation strategies
The energy demands associated with AI training, data processing, and operating IoT devices and UDT systems can lead to heightened carbon footprints and strain energy resources. These processes often require significant computational power, which consumes considerable electricity.	Implement energy-efficient algorithms and hardware, invest in renewable energy sources, promote green computing, and optimize data center cooling systems.
The rapid obsolescence of technology components and devices of AIoT and UDT can result in the accumulation of e-waste, posing challenges for proper disposal and recycling.	Promote product durability, encourage recycling and reuse programs, and advocate for the development of sustainable and modular device designs.
The production and disposal of hardware components for AI, AIoT, and UDT devices and systems contribute to resource depletion, impacting ecosystems and biodiversity.	Adopt circular economy practices, utilize eco-friendly materials, and explore alternative and sustainable sourcing options for technology components.
Inadequate management of e-waste may lead to the release of harmful substances into the environment, posing risks to soil, water, and air quality.	Strengthen e-waste management systems, enforce responsible disposal practices, and invest in research for eco-friendly electronic materials.
Large-scale data centers, crucial for AI, AIoT, and UDT operations, can have substantial environmental footprints, including water usage, land use, and CO_2_ emissions.	Utilize energy-efficient data center designs, explore decentralized computing models, and prioritize the use of renewable energy sources for data center operations.
The deployment of IoT sensors and infrastructure may affect local ecosystems, thereby potentially disrupting natural habitats and biodiversity in urban areas.	Implement strategies that consider biodiversity and ecosystem preservation, and integrate green infrastructure
Integration of UDT in management and planning systems may lead to various environmental impacts, including also potential disruption of urban ecosystems and vast electricity demand due to tremendous calculations for data-driven AI models and massive IoT networks envisioned.	Employ sustainable UDT design principles, consider the life cycle of UDT components, conduct thorough environmental impact assessments for UDT projects, and explore energy-efficient computation methods for UDT processes.

Integrating AI, AIoT, and UDT brings forth a spectrum of environmental challenges that necessitate careful consideration. Balancing technological innovation's benefits with environmental quality preservation requires a concerted effort to mitigate these risks. Stakeholders in urban development must navigate these challenges with a commitment to minimizing the ecological footprint of advanced technologies. This requires carefully designed strategies and best practices to foster a harmonious balance between urban technological landscapes and environmental well-being.

#### 3.4.6 Resource allocation and financial constraints

Implementing and sustaining AI/AIoT-driven UDT in sustainable smart cities demands substantial technological infrastructure deployment, operation, and maintenance investments. In addition to the substantial initial development costs associated with implementing this large-scale system, there are significant expenses for operation and maintenance to ensure reliability and meet real-time expectations. However, despite these investments, the long-term cost-effectiveness of UDT remains to be fully substantiated [28]. The challenges associated with resource allocation and funding mechanisms represent formidable obstacles to realizing the transformative potential of UDT. The financial commitments required for deploying the requisite sensor networks for reliable real-time data collection, data storage facilities, computation resources, and licensing commercial platforms are considerable and often exceed the budgetary capacities of sustainable smart city planning initiatives. The exploitation of the full potential of the technical components of UDT may be constrained by financial considerations, necessitating a careful balance between costs and functionality [203,204]. Therefore, governments, municipal bodies, and private entities face critical decisions on allocating resources more effectively to develop and implement UDT in pursuing sustainable smart urban development. Furthermore, UDT's continuous maintenance, updating, and expansion add to the financial burden over time. Indeed, the most efficient UDT systems entail higher initial investment costs and possess greater technical complexity compared to established conventional planning practices, rendering their promotion and acceptance a challenging task [55,198,204]. The costs associated with the utilization and maintenance of the system are also substantial [179].

Addressing these challenges requires a strategic approach to financial planning and recognizing the long-term benefits that UDT can bring to sustainable urban development. Collaboration between the public and private sectors and innovative financing models could be crucial in overcoming financial barriers. Sustainable funding mechanisms must be devised to ensure the long-term viability of UDT initiatives, fostering a commitment to ongoing innovation, resilience, and adaptability in sustainable smart cities. Moreover, the initiatives that promote knowledge-sharing and capacity-building can empower local governments and municipalities to make informed decisions about resource allocation for UDT projects. However, resource constraints and disparities in access to UDT infrastructure can exacerbate inequalities in urban communities, highlighting the need for inclusive and equitable deployment strategies. Addressing this challenge requires a holistic and collaborative approach involving multidisciplinary stakeholders to ensure UDT-driven urban planning initiatives’ effective and sustainable implementation. By navigating these challenges effectively, sustainable smart cities can unlock the full potential of AI/AIoT-driven UDT to create resilient, efficient, and environmentally sustainable cities.

#### 3.4.7 Community engagement and inclusivity

Ensuring that AI/AIoT-driven UDT contributes to inclusive urban development and addresses the needs of diverse communities is a multifaceted challenge that extends beyond technological considerations. It necessitates a comprehensive approach that includes robust community engagement strategies. Without careful planning and intentional efforts, there is a risk that sustainable smart city initiatives, particularly those considering the implementation of UDT, may inadvertently perpetuate or exacerbate existing socioeconomic disparities and social inequalities. The concept of a people-centric UDT seeks to recenter socioeconomic aspects at the forefront of the discourse. This vision advocates that UDT should prioritize enhancing the quality of life for all citizens rather than solely pursuing economic efficiency [59]. Among the current hurdles to achieving inclusivity are the bias of developers' socio-economic background, contributing to a deficiency in cultural diversity in data [25] and the selection of information communication methods [59]. Researchers highlight a notable gap in effective citizen and community engagement methods and an unclear understanding of the benefits of UDT among citizens [[25], [28], [170], [171]]. One of the key aspects of addressing this challenge is to prioritize inclusive community engagement throughout the entire lifecycle of UDT projects in sustainable smart cities. This involves establishing mechanisms for soliciting input from diverse community members, including those from marginalized or underrepresented groups [205]. Community-based participatory approaches can empower residents to actively contribute their perspectives, expectations, needs, and concerns, fostering a sense of ownership in the decision-making processes related to urban planning and development [206].

Effective community engagement is about information dissemination and creating avenues for meaningful dialogue and collaboration. It requires a shift towards co-creation, where community members become active partners in shaping UDT's goals, features, and deployment strategies [207]. Additionally, urban planners and policymakers must consider the accessibility of digital technologies, ensuring that the benefits of UDT reach all citizens and communities, regardless of their socioeconomic status or technological literacy. Charitonidou [25] explored the impact of the virtual public sphere on urban experiences in an emerging data-driven society, focusing on urban-scale DT — a tool for simulating urban environments and developing scenarios for policy problems. The author examines the shift from technical to socio-technical perspectives in smart cities, contending that, despite the intention of UDT to improve citizen participation in decision-making related to urban planning, concerns arise due to its reliance on a limited set of variables and processes. The study provides useful insights into the tension between reality and idealism in abstracting variables in urban scale DT, revealing challenges in this abstraction process. Regardless, the socio-cultural context of different communities influences the acceptance and adoption of UDT and other technology-driven solutions, necessitating tailored approaches to stakeholder engagement and communication.

Overall, while technological advancements in AI, AIoT, and UDT offer immense potential for revolutionizing data-driven environmental planning in urban contexts, it is imperative to recognize that technical solutions do not solely determine urban sustainability. Rather, it is a multifaceted concept encompassing socio-economic and cultural dimensions, which play pivotal roles in shaping the livability and inclusivity of cities. Neglecting these dimensions of urban sustainability can have profound implications for urban communities. For instance, urban planning and development projects that prioritize technological solutions without considering the needs and preferences of diverse social groups risk exacerbating existing inequalities. Moreover, a narrow focus on efficiency and optimization may inadvertently prioritize economic interests at the expense of social equity and cultural preservation. For example, implementing AI/AIoT-driven UDT projects that optimize resource allocation and enhance operational efficiency may inadvertently overlook marginalized communities’ access to essential services. Therefore, an inclusive approach to urban sustainability requires a holistic understanding of the socio-economic and cultural contexts in which technological solutions are implemented. This entails engaging with local communities, understanding their needs and aspirations, and incorporating their perspectives into the planning and decision-making processes. By adopting a more inclusive and participatory approach, urban planners and policymakers can ensure that technological advancements in AI, AIoT, and UDT are leveraged to promote social equity, cultural diversity, and sustainable city development.

In conclusion, this study has examined the intricate interplay of AI, AIoT, UDT, data-driven urban planning, and environmental sustainability through conceptual and thematic categories. Each category serves as a focused lens through which the multifaceted dimensions of the topic have been analyzed, drawing insights from various studies to offer a well-rounded perspective. This deliberate organization has not only enhanced the clarity and coherence of the study but has also facilitated a more profound understanding of the synergies, challenges, and potential pathways forward in this burgeoning field. This study lays the groundwork for further research and practical implementations to foster sustainable smart urban environments guided by innovative computational technologies and models.

## 4 Discussion

The discussion section provides a critical analysis and interpretation of the findings derived from the study, focusing on elucidating the relevance of the results in the broader context of the research domain. It entails interpreting the findings, comparing them with the existing literature to contextualize their significance, and addressing their practical and theoretical implications in advancing knowledge. Additionally, it acknowledges any limitations of the study and discusses their potential impact on the interpretation of results to ensure a balanced assessment. Finally, suggestions for future research directions are proposed based on the insights gained from the current study, guiding the trajectory of further investigations in the field.

### 4.1 Interpretation of results

In response to RQ1, the study elucidates the theoretical and practical underpinnings of the convergence of AI, AIoT, UDT, data-driven planning, and environmental sustainability in the context of sustainable smart cities. These foundational components are rooted in a multidisciplinary framework emphasizing interdisciplinary and transdisciplinary approaches. Their integration involves leveraging advanced technologies to inform data-driven decision-making processes in sustainable urban development practices. The study identified and forged these components essential for synergistically integrating AI, AIoT, and UDT to advance data-driven environmental planning in sustainable smart cities. These components' complex yet interconnected nature underscores the need for holistic strategies in sustainable urban development.

Transitioning to RQ2, the findings indicated that integrating AI and AIoT technologies brings about a fundamental shift, reshaping the landscape of urban planning and unveiling innovative pathways to enhance the environmental performance of sustainable smart cities. By utilizing AI and IoT technologies and combining their capabilities, these cities can gather real-time environmental data, analyze trends, and optimize resource allocation to mitigate environmental impacts and foster sustainability. This underscores the potential of technology- and data-driven solutions in addressing complex environmental challenges.

Moving forward to RQ3, the study demonstrated that AI and AIoT have the potential to augment the capabilities of UDT, enabling advancements in data-driven environmental planning processes in sustainable smart cities. By integrating AI-driven analytics and IoT-generated data into UDT platforms, these cities can simulate various environmental scenarios, assess the impact of urban development projects, and optimize infrastructure designs to enhance environmental sustainability. This highlights the synergistic relationship between emerging technologies and environmental urban planning methodologies.

Finally, in addressing RQ4, the study uncovered a complex terrain marked by various challenges and barriers that arise in the integration and implementation of AI, AIoT, and UDT in data-driven environmental planning processes in sustainable smart cities. These include privacy and security concerns, ethical and social issues, lack of data interoperability, environmental risks, financial constraints, regulatory inadequacies, lack of community engagement, and stakeholder conflicts. Surmounting or mitigating these challenges and barriers requires concerted efforts and robust strategies. These involve implementing robust privacy and security measures for data protection, addressing ethical and social concerns through transparent and inclusive decision-making processes, developing standardization protocols and frameworks, sustainable technology and eco-design practices, securing funding and resources, and devising sustainable funding schemes, advocating for robust data governance frameworks that support innovation while safeguarding public interests, and facilitating stakeholder collaboration and conflict resolution through effective communication and engagement and capacity-building endeavors. This highlights the importance of addressing socio-technical challenges to effectively deploy technology- and data-driven solutions in urban contexts.

### 4.2 Comparative analysis

The systematic review represents a significant advancement in research and practice by addressing a conspicuous gap in the existing literature. While prior review studies on sustainable smart cities have predominantly focused on isolated components such as AI [[22], [23], [24]], AIoT [7,8], and UDT [28,208], they have often overlooked the convergence of these technological elements and its significant impact on data-driven environmental urban planning. To put it differently, the intersection of advanced technologies and collaborative models in the context of sustainable smart cities has received limited attention regarding their data-driven approaches to environmental planning. Specifically, the intricate interplay and potential synergies among AI, AIoT, and UDT functionalities to advance data-driven environmental planning processes have thus far remained underexplored, if not entirely neglected. This study fills a crucial void in the literature by conducting a comprehensive systematic analysis of these interconnections. It transcends traditional approaches by offering a holistic perspective that sheds light on the complex relationships, nuanced dynamics, and untapped potentials associated with emerging sustainable smart cities. Moreover, the critical insights presented in this systematic review challenge the prevailing paradigm of studying isolated components, advocating instead for an integrated and interdisciplinary research approach in sustainable smart urban development.

Furthermore, it becomes evident that while several studies have recently begun to address the intersection between AI and AIoT technologies and urban planning practices [1,3,[4], [6]], environmental sustainability has received comparatively less attention. This disparity is also noticeable in studies focusing on the integration of AI and AIoT with UDT [30,165]. While these studies have made strides in understanding the potential of these advanced technologies from various perspectives, there remains a significant gap in comprehensively addressing environmental sustainability within the framework of sustainable smart cities. This highlights the need for further attention towards integrating environmental sustainability considerations into developing and implementing AI and AIoT-driven UDT solutions for data-driven urban planning and design.

### 4.3 Implications for research, practice, and policy making

The study holds profound implications across three crucial dimensions: research, practice, and policymaking. In the realm of research, scholars and researchers can leverage the conceptual and theoretical insights gained from this study to advance further investigations into the synergistic integration of AI, AIoT, and UDT for driving sustainable urban development forward, focusing on data-driven environmental planning. Additionally, the study underscores the potential for fostering interdisciplinary collaboration, encouraging scholars from diverse fields to join forces in exploring the complex dynamics of sustainable smart urban development. This collaborative approach contributes to a more holistic understanding of this field and enriches the discourse on technological integration and interdisciplinary convergence.

In urban planning, practitioners stand to gain significant advantages from the redefined landscape influenced by AI, AIoT, and UDT technologies. Acquiring actionable insights, these practitioners can leverage technological advancements to enhance environmental sustainability practices and implement targeted interventions for more resilient and environmentally focused urban development. Additionally, the study imparts valuable perspectives on the ethical and social considerations associated with AI, AIoT, and UDT, promoting the development of responsible and inclusive urban planning strategies. This multifaceted approach equips practitioners with a holistic view, allowing them to comprehend the complexities of urban environments while prioritizing sustainability, resilience, and the well-being of communities. Furthermore, practitioners can make more informed decisions based on real-time data and predictive analytics by leveraging AI, AIoT, and UDT technologies. This improves the efficiency and effectiveness of data-driven environmental urban planning initiatives and facilitates adaptive and proactive strategies to address emerging challenges and opportunities in sustainable urban development.

In the policy domain, policymakers are presented with a transformative opportunity to incorporate the insights gleaned from this study into strategic initiatives for sustainable smart urban development. This knowledge enables them to formulate effective policies that advance sustainable smart cities and align with the synergistic integration of AI, AIoT, and UDT technologies, addressing key challenges and promoting environmentally conscious urban planning. Moreover, policymakers can leverage this knowledge to develop regulations and governance frameworks that promote these technologies' ethical and responsible use and encourage inclusive approaches for sustainable smart cities. The study contributes to shaping policies that not only navigate the complexities of emerging technologies but also prioritize the long-term well-being of urban communities and the preservation of the environment.

### 4.4 Limitations

While this comprehensive systematic review contributes significantly to understanding the convergence of AI and AIoT, UDT, urban planning, and environmental sustainability, it is still important to acknowledge certain limitations. One inherent limitation lies in the rapidly evolving nature of AI, AIoT, and UDT technologies, which may introduce new dimensions and considerations not captured in the analysis and synthesis of the selected studies. Moreover, the study's scope is confined to the existing body of literature, potentially excluding emerging trends or perspectives. Additionally, synthesizing diverse research findings involves some abstraction, which might oversimplify nuances present in individual studies. Furthermore, the temporal constraint, covering studies from January 2019 to December 2023, may omit recent developments in the field. Also, the review process may introduce biases, and the inclusion criteria might exclude relevant studies that do not precisely align with predefined parameters. Lastly, the interpretation of findings may be influenced by the researchers' perspectives, leading potentially to subjective judgments. The study recognizes that potential biases in the review process and the interpretation of findings are intrinsic to any comprehensive analysis and synthesis of existing literature. While these constraints are acknowledged, the study underscores the need for ongoing research to capture recent developments, refine methodologies, and address potential biases. As the landscape of sustainable smart cities continues to evolve, future research endeavors can build upon these acknowledged limitations to further enrich the understanding of the complex dynamics in this interdisciplinary field.

### 4.5 Recommendations for future research

As the comprehensive systematic review explores the multifaceted interplay of AI, AIoT, UDT, data-driven urban planning, and environmental sustainability in the dynamic landscape of sustainable smart cities, it reveals a terrain ripe with potential for future research endeavors. By pinpointing critical gaps and their implications, this subsection outlines a roadmap for advancing sustainable smart cities (Table 4). These identified gaps pave the way for strategic initiatives aimed at propelling sustainable smart urban development forward, while the recommendations serve as a cornerstone for further investigations into the transformative potential at the nexus of data-driven technology, urban planning, and environmental sustainability.

**Table 4:** Research gaps, implications, and corresponding future research.

Research gaps	Implications	Corresponding future research
Data privacy and security in AI, AIoT, and UDT applications	Safeguarding sensitive information and ensuring citizen privacy are imperative for building trust in sustainable smart city technologies.	Investigate robust measures and mechanisms for data security and privacy in AI, AIoT, and UDT applications and propose policy frameworks to protect user personal and sensitive data in data-driven urban planning initiatives.
Ethical and societal considerations in AI, AIoT, and UDT integration in urban planning.	Ensuring responsible and equitable technology deployment, addressing potential biases, and safeguarding societal values in urban development.	Conduct in-depth studies on the ethical and societal implications of integrating AI, AIoT, and UDT in data-driven urban planning; investigate relevant frameworks emphasizing fairness, transparency, and accountability; and develop frameworks for human-centric decision-making and evaluate their impact on diverse communities and citizens.
Interoperability obstacles in sustainable smart city ecosystem.	Hindering seamless communication and coordination among diverse technologies and systems in AI/AIoT-driven UDT.	Investigate standardized protocols and interfaces to enhance AI/AIoT-driven UDT interoperability, assess the effectiveness of interoperable interfaces in diverse urban contexts, and promote a cohesive and integrated sustainable smart city ecosystem.
Long-term environmental impacts of integrating AI, AIoT, and UDT in urban planning.	Uncovering the environmental costs and risks of ongoing and future AI, AIoT, and UDT in data-driven urban planning initiatives is essential for long-term goals of sustainability.	Undertake comprehensive studies assessing the long-term environmental implications of AI, AIoT, and UDT applications in data-driven urban planning, considering factors, such as energy consumption, resource depletion, water usage, e-waste, and overall ecological footprint, as well as develop solutions and strategies promoting sustainable and eco-design principles and green computing approaches.
Community engagement and inclusivity.	Ensuring active participation and representation of diverse communities in AI/AIoT-driven UDT is fundamental for equitable and inclusive urban development.	Explore methods for enhancing community participation in AI/AIoT-driven UDT initiatives, develop strategies for inclusivity in data-driven decision-making processes in urban planning, and assess the impact of innovative technologies on marginalized communities.
Financial capital and funding.	Adequate financial resources are essential for implementing and scaling AI/AIoT-driven UDT in sustainable smart city initiatives.	Examine funding models for AI/AIoT-driven UDT initiatives; explore alternative funding models, public-private partnerships, and innovative financing mechanisms to support UDT projects; and assess the economic feasibility of these projects and their cost-effectiveness in the long run.
AI, AIoT, UDT, and data-driven urban planning integration	Seamlessly integrating AI, AIoT, UDT, and urban planning requires overcoming various technical and logistical challenges.	Investigate strategies and frameworks to overcome integration challenges, promote a cohesive and interconnected urban ecosystems, and evaluate the effectiveness of integrated systems in real-world urban contexts by conducing case studies and applying best practices.
AI and AIoT governance and regulatory frameworks	Establishing governance and regulatory frameworks is crucial for responsible and ethical use of AI and AIoT in urban planning.	Investigate existing AI and AIoT governance models; propose regulatory frameworks for AI and AIoT applications in urban planning contexts; and formulate governance frameworks and regulations to guide the ethical deployment of AI and AIoT in urban planning.

This comprehensive set of recommendations for future research emerges from an in-depth analysis of the challenges and obstacles inherent in the dynamic interplay of AI, AIoT, UDT, data-driven urban planning, and environmental sustainability. The proposed research avenues aspire not only to fill existing gaps but also to pave the way for a more inclusive, safe, and environmentally sustainable urban future. Collaborative efforts across disciplines involving researchers, urban planners, technologists, and policymakers are essential to collectively investigate these unexplored territories and create resilient and equitable sustainable smart cities.

Furthermore, future research endeavors can address the identified limitations to enhance the robustness and comprehensiveness of systematic reviews in this domain. Firstly, researchers can adopt more flexible methodologies that accommodate the dynamic nature of AI, AIoT, and UDT technologies, allowing for real-time updates and the inclusion of emerging trends. Additionally, expanding the temporal scope beyond the predefined timeframe can capture recent developments and ensure the currency of the literature synthesis. Furthermore, efforts can be made to mitigate biases introduced during the review process by employing rigorous approaches to study selection and data analysis. Embracing interdisciplinary collaboration and incorporating diverse perspectives can enrich the interpretation of findings and mitigate the risk of subjective judgments. Moreover, future research can explore innovative methodologies for synthesizing heterogeneous data sources and capturing nuanced insights from individual studies. By addressing these avenues, future research can advance knowledge and understanding of sustainable smart cities, laying the foundation for informed decision-making and resilient urban development strategies.

## 5 Conclusion

As AI, AIoT, and UDT increasingly permeate urban landscapes, a profound and nuanced understanding of the implications of their incorporation into data-driven environmental planning processes to advance sustainable urban development practices becomes imperative. In addressing the challenges and complexities of urban planning and environmental sustainability, the call to embrace and leverage integrated and holistic data-driven solutions has never been more emphasized in the dynamic landscape of sustainable smart cities. This underscores the critical importance and relevance of embarking on a thorough exploration — a comprehensive analysis and synthesis of various research strands — with the primary objective of generating cohesive new insights and perspectives.

This comprehensive systematic review addressed a critical knowledge gap. This involved uncovering the intricate interplay of AI, AIoT, UDT, data-driven urban planning, and environmental sustainability and elucidating the nuanced dynamics and untapped synergies in the complex ecosystem of sustainable smart cities. Guided by the formulated research questions, the findings of this comprehensive systematic review provided fertile insights into the theoretical foundations, practical applications, and anticipated challenges associated with the convergence of these technologies, models, and domains. Specifically, they surpassed mere interdisciplinary theoretical enrichment, offering valuable insights into the transformative potential of integrating AI, AIoT, and UDT technologies to advance sustainable urban development practices. By enhancing data-driven environmental planning processes, these integrated technologies and models offer innovative solutions to address complex environmental challenges. However, this endeavor is associated with formidable challenges and complexities that must be carefully navigated and overcome to achieve desired outcomes.

The contributions of this study resonate within academic discourse and practical knowledge. By synthesizing diverse perspectives, resolving inconsistencies, harnessing synergies, identifying challenges and barriers, acknowledging limitations, spotting research gaps, and providing forward-looking suggestions for future research endeavors, the study offers a comprehensive understanding of the symbiotic relationship and collaborative interaction among AI, AIoT, UDT, urban planning, and environmental sustainability. These insights have profound implications for researchers, practitioners, and policymakers, providing a roadmap for fostering resiliently designed, technologically advanced, and environmentally conscious urban environments.

## CRediT authorship contribution statement

**Simon Elias Bibri:** Conceptualization, Methodology, Investigation, Data Curation, Visualization, Software, Writing - Original Draft, Writing - Review & Editing. **Jeffrey Huang:** Conceptualization, Writing - Review & Editing. **Senthil Kumar Jagatheesaperumal:** Investigation, Visualization. **John Krogstie:** Writing - Review & Editing.

## Declaration of competing interest

The authors declare that they have no known competing financial interests or personal relationships that could have appeared to influence the work reported in this paper.
